# Influence of Carbon Dioxide and pH on Influenza Virus in Sessile Saliva Droplets

**DOI:** 10.1021/acs.est.5c12672

**Published:** 2026-02-26

**Authors:** Alexandra K. Longest, Sonali Srivastava, Frank A. Mazzola, Rania E. Smeltz, Jeffrey L. Parks, Liviana K. Klein, Nisha K. Duggal, Peter J. Vikesland, Linsey C. Marr

**Affiliations:** 1 Department of Civil and Environmental Engineering, 1757 Virginia Tech, Blacksburg, Virginia 24060, United States; 2 Department of Biomedical Sciences and Pathology, Virginia-Maryland College of Veterinary Medicine, Virginia Tech, Blacksburg, Virginia 24060, United States

**Keywords:** inactivation, decay, humidity, Raman, acidic, buffer, air, aerosol

## Abstract

Upon exhalation, virus-laden respiratory droplets experience rapid changes in environmental conditions that lead to chemical and physical alterations that can affect virus infectivity. By manipulating the concentration of gaseous carbon dioxide (CO_2_) surrounding sessile saliva droplets, we altered their chemistry and then assessed the impacts of these changes on the infectivity of influenza A virus at relative humidities of 30, 50, and 80%. For virus exposed to low CO_2_ (<0.005% CO_2_ in N_2_) vs high CO_2_ (4.3–5% CO_2_ in N_2_), differences in inactivation were small except at 80% RH, where the virus decayed less (i.e., maintained greater infectivity) in low CO_2_ than in high CO_2_. The difference exceeded 1log_10_ at 2 h. For comparison, virus inactivation in ambient air (0.04% CO_2_) varied across conditions, sometimes exceeding and sometimes falling below that observed under high- and low-CO_2_ atmospheres. Collectively, these results suggest that the driving factors for virus inactivation vary with RH. We measured droplet pH using gold nanoprobes in combination with surface-enhanced Raman spectroscopy and found that pH increased in low CO_2_ and decreased in high CO_2_ at 80% RH by ∼1 pH unit in both cases. Results were consistent with chemical equilibrium modeling, which indicated that both carbonate and phosphate buffering were important. Changes in pH were smaller or insignificant at 30 and 55% RH. At these low and medium RHs, rapid evaporation of water from the droplets and the resulting increase in viscosity may limit changes in pH. Measured changes in pH did not appear to be sufficient to drive virus inactivation under any tested condition. This finding suggests that pH likely does not impact influenza transmission by fomites.

## Graphical abstract

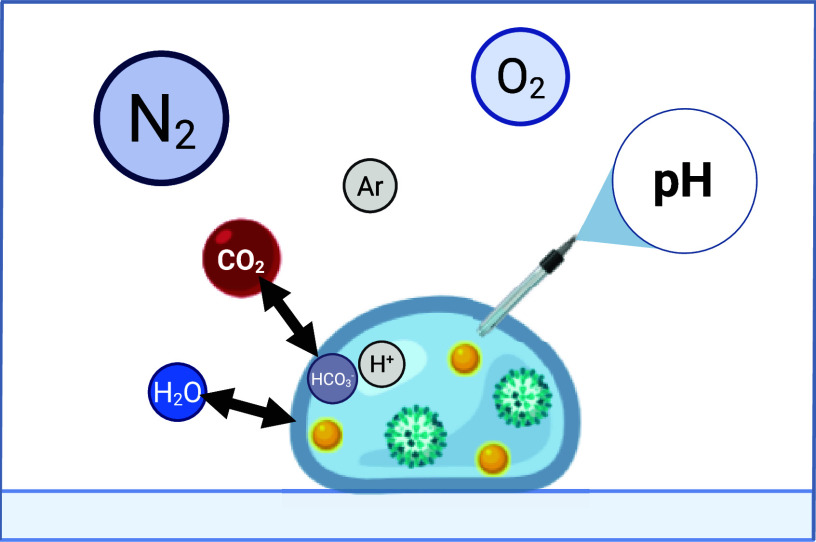

## Introduction

Efficient transmission of respiratory viruses through the air and on surfaces depends on many factors, including their environmental stability. A mechanistic understanding of virus inactivation within respiratory particles (i.e., droplets of all sizes, including aerosols and deposited droplets, and different evaporation stages) remains elusive because of the complexity of the system and its many interrelated factors, including the physical and chemical properties of the particle, relative humidity (RH), temperature, atmospheric composition, and pH, as well as virus structure and type.^1^ Respiratory droplets vary in composition and size depending on host factors and the site of origin within the respiratory tract.^2,3^

Respiratory droplets, produced either deep in the lungs or more proximal to the mouth or nose, are a complex aqueous mixture of organic macromolecules and inorganic salts. Saliva, for example, contains monovalent, divalent, and trivalent salts of carbonate, phosphate, sulfate, and chloride in addition to carbonaceous material.^4^ Upon exhalation indoors, droplets experience a rapid change in environmental conditions as they transit from approximately 37 °C and 100% RH inside the respiratory tract to room temperature and lower RH in indoor air.^5,6^ Due to the change in RH, the water in the droplets begins to evaporate and solute concentrations increase, often to the point of precipitation or phase separation. The final physical and chemical state is dependent on the surrounding RH, the initial droplet size, and the starting droplet composition.^1,7–9^ As described in Huynh et al.,^7^ three final states are possible: (1) *fluid-* or *liquid-like* with decreased viscosity, increased solute concentrations, and protein aggregate formation, (2) *multiphase semisolid* with increased viscosity due to thickening, gelation, and aggregation of organics and inorganics, and (3) *partially crystalline solid*, with crystallized salts and amorphous solids that significantly hinder diffusion.^1,7^ The solid state is not necessarily void of water since the droplets contain hygroscopic organics and salts that have not crystallized.^9^ Rockey et al.^10^ presents images showing the different states of 1 μL saliva droplets after 2 h. At 20% RH, droplets effloresced, forming densely packed crystalline structures. At 50% RH, droplets exhibited a less dense crystalline structure, suggesting a semisolid state in which organics within the droplets inhibited crystalline growth. At 80% RH, the droplets never fully dried, but became gelatinous with small aggregates.

Yang et al.^11^ and Luo et al.^9^ suggest that evaporation and condensation could affect droplet pH, leading to conformational changes in virus surface glycoproteins and virus inactivation.^12^ Upon expulsion, respiratory droplets experience a shift in ambient carbon dioxide (CO_2_) levels from 4 to 5%, (40,000–50,000 ppmv) within the respiratory tract to approximately 0.04% (400 ppmv) in the environment.^8^ Respiratory fluids contain bicarbonate at concentrations of 0.06–3.66 g/L.^13^ When droplets are expelled into ambient air, CO_2_ partitions out of the droplets to re-establish chemical equilibrium.^8,9,14^ In isolation, this process would cause droplet pH to rise.^15^ However, the presence of other constituents in saliva such as phosphate and organic macromolecules that also contribute to saliva alkalinity and buffer capacity complicate this interpretation. Further, the surrounding air may contain trace gases, such as nitric acid, that can condense into and acidify the particles.^9,12^ Changes in pH have been suggested to drive virus inactivation in respiratory particles,^8,9,11,14^ but such changes have not been experimentally confirmed in real respiratory fluid due to the challenge of measuring pH in small droplets and aerosol particles.^1^

The capacity for air to inactivate airborne pathogens was first described as the “open air factor” in 1968.^16^ Ambient air consists of 78% nitrogen (N_2_), 21% oxygen (O_2_), 0.9% argon, 0.04% CO_2,_ and other trace gases. Hydrogen peroxide, ozone, and chlorinated gases are recognized virucides and bactericides and are commonly used in disinfection.^17^ However, the number of studies on the impact of other naturally occurring constituents of air is limited. Cox and Hess observed increased survival of Gram-negative bacteria in the absence of O_2_.^18–20^ Prior studies with viruses have suggested that their survival does not differ between exposure to ambient air vs pure N_2_.^14,21–23^ Haddrell et al.^14^ observed increased stability of the Delta variant of SARS-CoV-2 at 0.08% CO_2_ (800 ppmv) compared to 0.05% CO_2_ (500 ppmv) in minimal essential medium (MEM) at 90% RH. They hypothesized that higher CO_2_ concentrations mitigated an increase in droplet pH, which in turn promoted virus stability.^14^ The cell culture media used by Haddrell et al.^14,23^ and others^21,22^ for inactivation studies may have similar general physicochemical properties as saliva,^14^ but the media do not replicate saliva’s complex chemistry. To date there have been no direct measurements of pH in saliva droplets and consideration of its effects on virus stability in this medium. Moreover, studies on the influence of air composition have predominantly focused on airborne particles rather than on deposited droplets. Fomites have the potential to be a substantial source of transmission, particularly in schools and daycare centers.^24^ Although influenza outbreaks occur annually and the virus’ persistence on surfaces has been previously investigated,^10,25–27^ the influence of CO_2_ concentration on virus decay on surfaces remains unexplored.

The objective of this study was to determine, as a function of RH, the effect of varying gas-phase composition on the inactivation of influenza virus suspended in saliva droplets. We examined influenza A virus (IAV) viability in sessile, 1 μL droplets on surfaces at low, medium, and high RH (30%, 55%, and 80%) in near-zero CO_2_ in N_2_, 5% CO_2_ (50,000 ppmv, similar to the level found in the respiratory tract) in N_2_, and ambient air containing 0.04% CO_2_ (400 ppmv) as a baseline. As CO_2_ is expected to affect droplet pH, we also evaluated alkalinity and the effect of buffering on virus inactivation. Finally, we characterized changes in droplet pH using gold nanoparticle pH nanoprobes and surface-enhanced Raman spectroscopy (SERS).^28,29^ Collectively, our results suggest that pH changes are not a major driver of IAV inactivation in sessile 1 μL saliva droplets.

## Materials and Methods

### Virus Stock and Quantification

Influenza virus A/California/07/2009 (H1N1pdm09) was used herein as previously described.^30^ Virus stock and Madin-Darby canine kidney (MDCK) cells were kindly provided by Dr. Seema Lakdawala (Emory University; Atlanta, GA). MDCK cells were grown at 37 °C in 5% CO_2_ in MEM (Thermo Scientific, Cat. No. 12360038) containing 10% fetal bovine serum (VWR, Cat. No. 97068–086), 1% penicillin-streptomycin (Thermo Scientific, Cat. No. 15240062), and 1% l-glutamine (Thermo Scientific, Cat. No. 25030081). IAV in MEM was propagated in MDCK cells for 48 h at 37 °C, and virus titers were measured using plaque assay on MDCK cells, following established methods,^25,31^ as described in the Supporting Information (SI). The initial titer was ∼10^6.5^ PFU/mL.

A subset of experiments was conducted using bacteriophage Phi6. Procedures for these experiments are described in the SI.

### Virus Inactivation in Varying Atmospheres

Experiments took place in a desiccator (Fisher Scientific) used as an atmospheric chamber, inside a biosafety cabinet supplied with HEPA-filtered air. IAV inactivation was measured in evaporating 1 μL saliva droplets at low, medium, and high RHs under two atmospheric compositions. Saturated salt solutions of potassium acetate, magnesium nitrate, and potassium chloride were used to target 30%, 55%, and 80% RH, respectively.^32^ We considered RH, a measure of water vapor, separately from other measures of atmospheric composition because of its influence on the evaporation rate of water from the droplets. The two atmospheres were ultrahigh purity N_2_ (Airgas, Lot No. 72–402796545–1) containing negligible CO_2_ and 5% CO_2_ and 95% N_2_ (Airgas, Lot No. 72–402880487–1). The latter CO_2_ concentration is similar to that in undiluted exhaled breath. Even though previous studies have demonstrated that O_2_ has no impact on virus decay,^21,23^ we excluded O_2_ from our experiments to focus on CO_2_ and isolate its effects on chemistry and virus inactivation. Ambient air, containing ∼0.04% CO_2_, O_2_, and other gases, was also tested to provide a baseline for comparison of results. Table 1 summarizes the composition of the two atmospheres, along with ambient air, and the nominal descriptors used throughout this paper. As described in the SI, sensors inside the chamber recorded RH, temperature, and CO_2_ concentrations (Figures S1 and S2).

**Table 1:** Composition of Three Experimental Atmospheres

	**dry percentage (%)** [Table-fn t1fn3]			
nominal descriptor	N_2_	O_2_	argon	CO_2_
low CO_2_ (<0.005% CO_2_ and 99.995% N_2_)	99.995	0.0001[Table-fn t1fn4]		<0.005
high CO_2_ (4.3–5% CO_2_ and >95% N_2_)	94.980			4.3–5
ambient air	78	21	0.9	0.04

a Trace gases such as ozone, nitric acid, ammonia, volatile organic compounds, and others may be present at a total concentration of <∼0.001%.

b According to the supplier, O_2_ may be present at a concentration up to 1 ppmv (0.0001%).

IAV stock, suspended in MEM, was diluted 1:10 into human saliva pooled from multiple donors (Innovative Research, Cat. No. IRHUSL5ML), and then ten 1 μL droplets, constituting a single sample, were pipetted onto a 6-well polystyrene plate (Corning, Cat. No. 07201588) in technical duplicates. The plate was immediately placed (<5 s) into the chamber for a specified time period. For low and high CO_2_ conditions, gas flowed from a cylinder into the chamber at 15 psi for 1.5 min before the valve was closed, and the droplets were then exposed to the selected atmosphere for 0.5, 1, 2, or 4 h. For medium and high RH, the gas was humidified before entering the chamber. Experiments for each time point were conducted sequentially and independently. Droplets were resuspended and collected using 500 μL of MEM. Previous studies have shown that this collection method efficiently recovers virus, with an average estimated recovery of 92% (ranging from 82 to 105%).^10,27^

For data points at 0 h, droplets were pipetted onto a plate in the biosafety cabinet and immediately resuspended to provide a starting titer and to account for any collection losses. Since IAV decays in bulk saliva over time, the virus was diluted into saliva aliquots just before the experiment began for each time point. IAV did not decay in bulk MEM over the duration of the experiment. Independent triplicate experiments for each atmospheric condition at each RH were performed.

### IAV Inactivation in Three Different Liquid Matrices in Bulk

The inactivation of IAV in bulk liquid (500 μL) was determined in three liquid matrices: artificial saliva (recipe provided Table S1), human saliva, and heat-inactivated human saliva. Preparation and collection details for the liquid matrices can be found in the SI. To heat-inactivate the human saliva, 1.5 mL of thawed saliva was incubated in a water bath at 80 °C for 20 min.^33,34^

IAV virus stock was diluted 1:10 into each matrix to reach 500 μL total volume in 500 μL vials (Genesee Scientific, Cat. No. 21–258) that minimized the headspace above the liquid. After being spiked with virus, vials were vortexed at medium intensity for ∼5 s. At 0 h, 50 μL of each suspension was diluted into 450 μL of MEM and stored at −80 °C until quantification. The vials containing the remaining 450 μL of each suspension were stored in the biosafety cabinet with the caps closed. After 4 h, 50 μL of each suspension was collected into 450 μL of MEM and stored at −80 °C until quantification. Each liquid matrix was tested in triplicate.

### pH Quantification Using SERS

The pH of evaporating saliva droplets was determined by adding gold nanoparticle pH nanoprobes to the droplets and then analyzing them using SERS (see SI).^29^ Briefly, gold nanoparticles were functionalized with pH sensitive 4-mercaptobenzoic acid (4-MBA). Changes in 4-MBA speciation alter the intensity of specific SERS bands that reflect the pH in the vicinity of the nanoprobes. The nanoprobes were suspended in MEM following synthesis. One-microliter droplets consisting of a volume ratio of 1:5 (nanoprobes in MEM):(human saliva) were deposited on quartz coverslips (Electron Microscopy Sciences, Cat. No. 72256–05) in a bag-like chamber (Atmosbag, Sigma-Aldrich, Cat. No. Z564397). A 1:10 dilution was not used because its signals were below the limit of detection. The polystyrene surface used for the virus inactivation experiments was incompatible because it generated background signals that interfered with the 4-MBA signal (Figure S3). The use of polystyrene did not substantially alter the measure droplet drying time (Figure S4). pH experiments were conducted using the atmospheric compositions described previously (Tables 1 and S2). Independent droplets were used for each time point and two independent replicate experiments were performed, with technical triplicates for each RH and atmospheric composition. Additional details about the experimental procedures, including pH calibration curves (Figure S5), are available in the SI. Because the droplets’ state evolves over time, separate pH calibration curves were developed for ‘wet’ and ‘dry’ droplets. The wet calibration curve was applied at 0 h for all conditions, whereas the dry calibration curve was used for data points collected at 1, 2, and 4 h at 80% RH and at 0.5, 1, 2, and 4 h at 30% and 55% RH. At 0.5 h for 80% RH, the droplet was in an intermediate state between wet and dry. The wet calibration curve was used, and the values derived from the dry calibration curve can be found in the SI (Figure S6) to show the range of possible pH defined by the fully wet and fully dry states. The potential effect of saliva concentration on both bulk and droplet pH was investigated by varying the saliva concentration and was found to be negligible (Figure S7).

### pH Modeling and Alkalinity

The inorganic composition of saliva was determined by inductively coupled plasma mass spectrometry (ICP-MS) using Standard Method 3125-B. The results (Table S3) were used as input for the equilibrium chemical speciation model MINEQL+ (Version 4.62.3). Model details and inputs are provided in the SI. Human saliva samples from three individuals were collected and tested in compliance with the guidelines of the Virginia Tech Institutional Review Board (approved protocol 24–594). Participants collected saliva in a 50 mL conical tube up to a final volume of ∼20 mL. These samples experienced varying degrees of CO_2_ loss, as would naturally occur with saliva droplets that deposit on surfaces. The total alkalinity of purchased human saliva and that from individual subjects was measured by acid titration (see SI). Total alkalinity (A_T_) is a measure of the capacity of saliva to neutralize acids and is defined as

$$A_{T}=\left[{\mathrm{OH}}^{-}\right]+\left[{{\mathrm{HCO}}_{3}}^{-}\right]+2[{{\mathrm{CO}}_{3}}^{2-}]+\left[{{\mathrm{HPO}}_{4}}^{2-}\right]+2[{{\mathrm{PO}}_{4}}^{3-}]-\left[H_{3}{\mathrm{PO}}_{4}\right]-\left[H^{+}\right]$$ 1

As indicated by eq 1, total alkalinity reflects the combined effects of carbonate and phosphate. Organic saliva constituents may also contribute alkalinity, but their concentrations are generally low relative to the carbonate and phosphate species and were therefore not considered. Some studies examining virus infectivity have used DMEM or MEM,^14,27,35^ whose alkalinities are higher than those reported for saliva. To assess the effect of alkalinity on virus inactivation, 200 μL of saliva was supplemented with 6.3 μL of 75 g/L sodium bicarbonate (NaHCO_3_; Gibco, Cat. No. 25080094) to match the total alkalinity of DMEM. Experimental details and environmental measurements (Figure S2) are provided in the SI.

### Statistical Analysis

All statistical analyses were conducted in R Studio version 2023.06.1. To examine the effect of gas-phase composition at each RH, a one-way ANOVA was conducted across atmospheric conditions at each time point, and pairwise comparisons were performed using Tukey’s honestly significant difference (HSD) test. Unpaired *t* tests were conducted between samples with and without NaHCO_3_ at each time point to test for significant differences. Unpaired *t* tests were also conducted to compare the initial droplet pH (at 0 h) with the pH at subsequent time points. An unpaired *t* test was performed to assess differences in IAV decay between 0 and 4 h in bulk liquid. An alpha level of 0.05 was used for all statistical tests.

## Results and Discussion

### Virus Inactivation in Different Atmospheres

We investigated the kinetics of IAV inactivation in 1 μL evaporating saliva droplets under atmospheres of low CO_2_ (<0.005 CO_2_ and >99.995% N_2_) and high CO_2_ (4.3–5% CO_2_ and >95% N_2_) at low (30%), medium (55%), and high (80%) RH. Ambient air provided a baseline for comparison. Figure 1 shows virus decay, or loss of infectivity, at 0.5, 1, 2, and 4 h. In all cases, decay increased with time and reached the limit of detection (i.e., no detectable infectious virus) after 2 h in some cases. In general, IAV decayed more rapidly at medium and high RH than at low RH.

**Figure 1:**
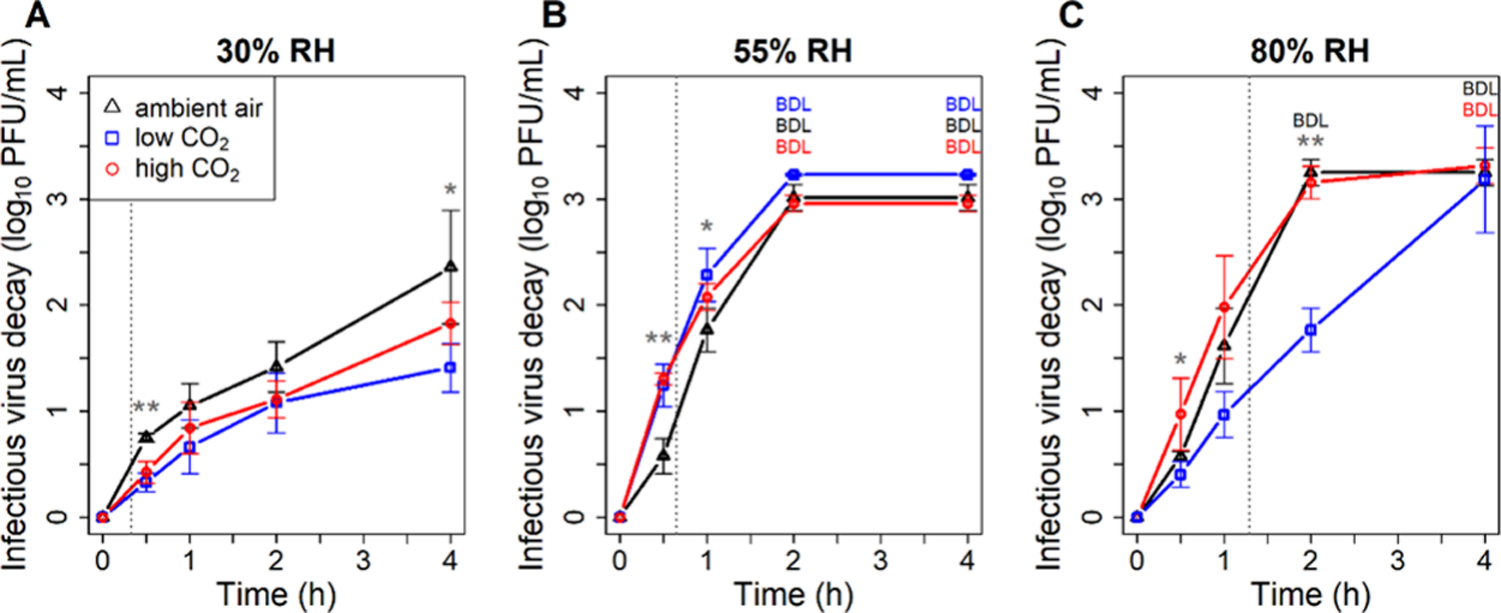
H1N1pdm09 IAV decay in 1 μL (initial volume), sessile, evaporating saliva droplets in ambient air (0.04% CO_2_), low CO_2_ (<0.005 CO_2_ and >99.995% N_2_), and high CO_2_ (4.3–5% CO_2_ and >95% N_2_) at (A) 30, (B) 55, and (C) 80% RH after 0, 0.5, 1, 2, and 4 h of exposure. Each point is the average ± standard deviation of three independent replicates. Asterisks indicate significant differences between atmospheric conditions, and the number of asterisks corresponds to the number of differences (i.e., the maximum possible number of asterisks is three, indicating that results at all atmospheric conditions are different from each other). BDL indicates that the virus titer was below the detection limit, so the decay was at least the value shown. Dotted, gray, vertical lines represent drying times (*t*_dry_) estimated using the approach of Rockey et al.^10^ as discussed in the SI and presented in Table S5.

At low RH (Figure 1A), we observed similar virus decay between low CO_2_ and high CO_2_, slightly less than in ambient air. Although the difference in decay was significant at 0.5 h, the magnitude of the difference was small and was within the day-to-day variability of the assay. Overall, the kinetics of virus decay were similar between the low and high CO_2_ atmospheres at low RH. At medium RH (Figure 1B), we observed 1.25log_10_, 1.31log_10_, 0.58log_10_, decay in low CO_2_, high CO_2_, and ambient air, respectively, at 0.5 h. Additionally, at 1 h, there was more decay in low CO_2_ (2.29log_10_) than ambient air (1.77log_10_), but the difference was small. As at low RH, the kinetics of virus decay were similar between low and high CO_2_ atmospheres with slight differences from the ambient air. At high RH (Figure 1C), IAV decayed significantly less in low CO_2_ compared to high CO_2_ at 0.5 and 2 h. At 2 h, the difference exceeded 1log_10_, and viable virus was detectable in low CO_2_ at 4 h, whereas the limit of detection was reached in the other atmospheres. Interestingly, similar decay was observed between high CO_2_ and ambient air at high RH, unlike at low and medium RHs.

Overall, the CO_2_ concentration had minimal effect on IAV inactivation in human saliva at low and medium RH but a larger effect at high RH. Results as a function of RH were consistent with those of previous studies.^27,30^ At high RH, IAV was more stable in low CO_2_ than in high CO_2_. We hypothesize that the overall decay kinetics as a function of RH and CO_2_ concentration are influenced by the droplet’s chemical composition. Yang et al.^36^ and Schaub et al.^26^ demonstrated that IAV inactivation varies with RH in 1 μL NaCl droplets due to differences in the salt concentration reached before efflorescence. In one study, the presence of sucrose, as a representative organic compound, decreased the NaCl molality during the drying phase and protected against NaCl-induced inactivation.^26^ These findings help explain the decay kinetics in our salt- and organic-containing saliva droplets. At low RH, the water in the droplets evaporated quickly (*t*_*dry*_ < 0.5 h) until efflorescence, resulting in only brief exposure to high concentrations of solutes^37^ and less IAV inactivation. IAV viability did not sharply decrease at efflorescence, potentially due to the protective effects of organic saliva constituents.^36^ At medium RH, evaporation was slower (0.5 h < *t*_*dry*_ < 1 h), leading to longer exposure to higher salt concentrations until efflorescence^37^ resulting in more decay compared than at low RH. At high RH, evaporation was even slower (1 h < *t*_*dry*_ < 2 h), and the salt concentration remained below saturation because the efflorescence RH was not reached.

Changes in the physical state of the droplets also help explain why the relationship between CO_2_ concentration and IAV inactivation varied with RH. At low and medium RHs, differences in IAV decay were relatively small across these atmospheric conditions. At high RH, IAV decay was significantly reduced in low CO_2_ compared to high CO_2_. These observations, coupled with recent reports in the literature,^1,7–9,26,27^ suggest that at low RH, the partially crystalline state of the droplet significantly slowed ion diffusion. This limited potential pH changes, resulting in minimal difference in virus infectivity as a function of atmospheric condition (Figure 1A). At medium RH, although evaporation was slower, the droplet’s microenvironment and physical properties, including its viscosity, changed with the onset of evaporation. Differences in ion diffusion rates within the droplets could have contributed to the difference in virus decay between conditions prior to efflorescence, but afterward, diffusion was hindered and virus decay converged (Figure 1B). We emphasize, however, that only one data point was available during the evaporating phase (0.5 h) at medium RH, so further investigation is needed. At high RH, the particles remained in a fluid-like and noncrystallized state that enabled CO_2_ to condense into the droplets and allowed fast ion diffusion within the droplet.

To contextualize our results, we tested the impact of different atmospheres (ambient air vs low CO_2_) on the inactivation of bacteriophage Phi6, a commonly used surrogate for IAV and other viral pathogens, suspended in DMEM (see SI for discussion; Figures S8-9). At low RH, the change from ambient air to low CO_2_ had no impact on virus decay (Figure S9A). At medium RH, Phi6 decayed less in low CO_2_ compared to ambient air at 2 and 4 h (Figure S9B). At high RH, we observed no loss in infectivity for Phi6 in low CO_2_ over 4 h, whereas there was approximately a 1log_10_ reduction in infectivity in ambient air at 4 h. Prior studies have shown Phi6 and IAV to decay at similar rates in 1 μL droplets.^26,27^ Here, however, we observed a significant difference in the decay kinetics for Phi6 (in DMEM) relative to IAV (in saliva), suggesting an influence of the suspension medium. Additional experiments with both sessile and levitated droplets are warranted, to better elucidate media effects on IAV infectivity.

### pH of Saliva Droplets in Different Atmospheres

We hypothesized that the change in virus inactivation as a function of RH and CO_2_ concentration may reflect changes in droplet pH. To test this hypothesis, we used pH nanoprobes and SERS to measure the pH of 1 μL saliva droplets under the same RH and atmospheric compositions as used in the virus inactivation experiments. Figure 2 shows pH evolution over time at RH values of 30%, 55%, and 80%. The pH of human saliva typically ranges from 6.2 to 7.6;^38^ however, due to methodological limitations, the initial pH of our bulk saliva was 8.5. We compared the pH changes across all atmospheres, assuming that CO_2_ was the only gas influencing the droplet pH and the O_2_ and other trace gases present would not impact pH.

**Figure 2:**
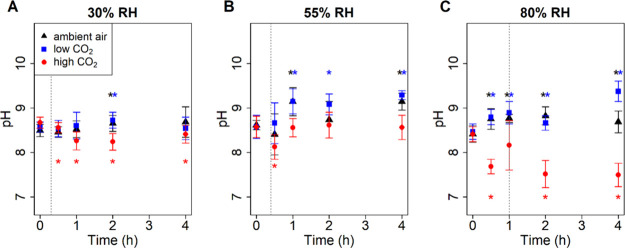
pH of 1 μL (initial volume), sessile, evaporating saliva droplets in ambient air (∼0.04% CO_2_), low CO_2_ (<0.005% CO_2_ and >99.995% N_2_), and high CO_2_ (4.3–5% CO_2_ and >95% N_2_) at (A) 30, (B) 55, and (C) 80% RH after 0, 0.5, 1, 2, and 4 h. Each point represents the average ± standard deviation of two independent replicates with three independent technical replicates. Asterisks indicate significant differences between the pH at 0 h and a subsequent time point. Dotted, gray, vertical lines represent droplet drying times on quartz (Figure S4B), which differ from the ones on polystyrene shown in Figure 1.

At low RH (30%), droplets reached a partially effloresced state at 0.33 h (Table S5).^10^ Under these conditions, the pH was constant in ambient air, except for a small increase (ΔpH ≈ +[0.15]) at 2 h compared to 0 h. In low CO_2_, the pH was higher at 2 h (ΔpH ≈ +[0.22]), but not at other times, compared to 0 h. In high CO_2_, the pH decreased (ΔpH ≈ −[0.4]) over 2 h. These results suggest that the pH remained relatively constant after efflorescence.

At medium RH (55%), droplets reached their quasi-equilibrium state at approximately 0.65 h (Table S5). Under ambient air and low CO_2_, the pH increased (ΔpH ≈ +[0.4–0.7]) at 1 h and then remained constant through 4 h (Figure 2B), suggesting that the pH was unchanged after the droplets reached quasi-equilibrium. This is likely due to the effloresced state of the particle slowing diffusion. Interestingly, under high CO_2_, the pH decreased (ΔpH ≈ −[0.4]) during the first 0.5 h before returning to the initial pH, a trend not observed in other conditions and currently unexplained.

At high RH (80%), the liquid-like state of the droplets allowed pH to change over the entire 4 h period, as shown in Figure 2C for low and high CO_2_ conditions. Under high CO_2_, the pH generally decreased over time, reaching a value 1 pH unit lower (ΔpH ≈ −[1.0]) at 4 h. Under low CO_2_, the pH increased over 4 h (ΔpH ≈ +[0.9]). In ambient air, pH changed minimally, possibly due to the prior degassing of CO_2_ due the freezing and thawing of the purchased saliva. The higher pH of our bulk saliva of compared to the normal pH for saliva^38^ may have affected the extent of pH change attributed to CO_2_ partitioning. However, as solely CO_2_ was driving the change in pH under low and high CO_2_ conditions, the final equilibrium pH of the droplets would be the same as if prior offgassing did not occur with our bulk saliva sample.

We measured the pH at the center and edge of our droplets and found no difference in the measured pH values. This result is consistent with Huang et al.^39^ for droplets containing a mixture of ammonium and phosphate salts, but is inconsistent with Wei et al.,^29^ who showed, at high RH, lower pH values at the edge of phosphate buffered droplets compared to the center. This latter result, corroborated by others,^40^ presumably reflects hydrogen ion accumulation at the air-particle interface. The complex composition of saliva, including proteins, surfactants, and other organics, may limit hydrogen ion accumulation at the air-particle interface, preventing formation of a pH gradient. Experiments to test this possibility were outside the scope of the present study.

Our results collectively indicate that, at low and medium RH, after the droplets effloresced, the pH remained constant, possibly reflecting low diffusivities in semisolid or solid particles. Our droplets are larger than those typically expelled, so the drying effect may be more or less limiting on the rate of pH change relative to smaller droplets, depending on the evolution of their physical state. However, to our knowledge, no studies have investigated how morphology changes in real respiratory droplets.

### Saliva pH and Alkalinity

The droplets remained liquid for an extended time at high RH, and we calculated the equilibrium pH as a function of the inorganic composition of saliva and the gas-phase CO_2_ concentration (Table S6 and detailed calculations provided in the SI). For this purpose, we used MINEQL+ and assumed an open system. This model suggested that the expected equilibrium pH for our droplets should range from 7.1 to 10.0 (Table S7), increasing as a function of the gas-phase CO_2_ concentration. These predicted pH values are all within 0.5 pH units of the pH values we measured at 4 h using the pH nanoprobes. When phosphate was not included in these calculations, the calculated equilibrium pH values were consistently higher.

The extent of pH change due to a shift in the gas-phase CO_2_ concentration is constrained by the total alkalinity of the system. The total alkalinity of the purchased saliva was 0.016 N, whereas that of saliva from three individual subjects ranged from 0.006 to 0.010 N (Table S8). We surmise that the higher alkalinity of the purchased saliva may result from the donors chewing paraffin wax to generate “stimulated” saliva, which is known to contain a higher concentration of bicarbonate compared to unstimulated saliva.^41,42^ Reported phosphate concentrations in saliva average 0.004 M (0.38 g/L), but can vary up to 6-fold.^43^ Based upon our ICP-MS measurements, the phosphate concentration in the purchased saliva was 0.00135 M (0.128 g/L) whereas that in a pooled sample from three subjects was 0.00224 M (0.212 g/L). These values suggest that on a molar basis alone, phosphate may contribute substantially to the total alkalinity. This inference is supported by MINEQL+ modeling, which suggests that phosphate contributes ∼8.3% of the total alkalinity at an equilibrium pH of 9.084 (Table S9).

Cell culture medium is frequently utilized to investigate virus inactivation.^44–46^ As shown in Table S8, however, the calculated total alkalinities of both DMEM and MEM greatly exceed those of saliva. The media have even greater buffer capacity than saliva. We sought to investigate the role of alkalinity in virus decay and thus added NaHCO_3_ to human saliva. As shown in Figure S10, the addition of NaHCO_3_, intended to better buffer changes in pH, did not affect virus decay at medium RH under low CO_2_ and only minimally affected it under high CO_2_. Augmenting the buffer capacity of saliva did not enhance IAV stability.

In saliva, total alkalinity is primarily defined by its carbonate and phosphate constituents. Organic compounds also contribute to the alkalinity but are generally at sufficiently low enough concentrations to be ignored.^41^ While a recent study of the pH of model respiratory fluid droplets primarily focused on carbonate,^47^ we postulate that both carbonate and phosphate should be considered when evaluating virus inactivation. Speciation of the inorganic carbon and inorganic phosphorus species, and their relative effects on buffer capacity, is sensitive to pH (eq 1). As shown in Figure S11, the calculated buffer capacity of saliva with a biologically reasonable total carbonate concentration ([CO_3_]_T_) of 0.01 M and a total phosphate concentration ([PO_4_]_T_) of 0.001 M is dominated by carbonate species for the pH ranges 4–6 and 9–11; for pH values between 6 and 9, both the carbonate and phosphate species contribute to the calculated buffer capacity. While neglecting the contribution of phosphate may be reasonable at lower and higher pH values, it should not be neglected for near-neutral pH conditions or for systems potentially subject to broad pH swings. This calculation highlights the importance of considering biological fluids. Simple, model systems can enable mechanistic insight into virus inactivation, but they do not fully address the complex chemistry of real respiratory droplets. Ultimately, results from both types of studies—those using model fluids and those using biological fluids—are needed to advance our understanding of virus inactivation.

### Relationship between pH and IAV Inactivation

Our results provide novel insights into the relationship between pH and virus infectivity. Previous research has shown that changes in pH can result in virus inactivation in a manner that differs between viruses.^9,48,49^ However, there have been no studies on the impact of pH on virus decay in deposited droplets. IAV is highly sensitive to acidic conditions (i.e., pH 5.5 or lower) due to the effect of pH on the conformation of viral surface proteins that dictate endosomal entry into cells.^9,12^ However, IAV is stable under alkaline conditions up to pH 11.^26,8,9^

We attempted to induce a larger shift than would be encountered in ambient air toward an alkaline pH by generating an environment with negligible CO_2_. In theory, this would promote CO_2_ offgassing and would generate a larger increase in pH than expected in ambient air. We also attempted to drive the pH in the opposite direction by exposing droplets to an atmosphere of 5% CO_2_ and 95% N_2_. Such conditions mimic the CO_2_ concentration within our respiratory tract as well as reduce the potential influence of other species such as trace gases. As shown in Figure 2, however, it was challenging to induce changes in droplet pH using either of these manipulations likely due to the buffer capacity of the droplets.

Our results suggest that changes in pH do not drive the virus inactivation observed within our system, suggesting that neither acidic nor alkaline pH changes appear likely to affect influenza transmission through fomites. Across all conditions, the pH in our droplets changed by 1 pH unit or less (Figure 2). While the extent of change of pH was limited by the initial offgassing of the saliva samples, the lowest and highest measured pH values (∼7.8 and 9.4 pH at low CO_2_ and high CO_2_, respectively) were not extreme enough to inactivate influenza virus.^26^ As our results demonstrate, pH changes much more under high RH conditions compared to low or medium RH. Accordingly, observations at high RH may not represent what occurs at low and medium RHs. Indoor RH typically ranges from 20 to 60% RH and thus our results suggest a pH change driven by CO_2_ may not be significant in indoor environments.

As our results suggest that pH does not drive IAV decay in sessile saliva droplets, the observation of significantly less decay in low CO_2_ compared to high CO_2_ at 80% RH (Figure 1C) cannot be explained through this mechanism. While this difference is intriguing, its underlying cause remains unknown. One hypothesis is that proteins or other antiviral components present in saliva^50^ are pH sensitive and could drive virus inactivation. Further investigation is needed to define the composition of human saliva and its effect on virus stability.

### Virus Inactivation in Bulk Human Saliva and Surrogate Fluids

Because changes in pH did not appear to drive virus inactivation in our system, we elected to investigate whether other saliva components (e.g., antiviral proteins including lysozyme and lactoferrin, sialic acids^51–53^ ) promote IAV inactivation. We compared IAV persistence in human and artificial saliva to assess the effect of respiratory fluid on virus decay in bulk. Figure 3 shows the viability of IAV in 500 μL of artificial saliva, human saliva, and heat-inactivated human saliva in sealed vials over 4 h at room temperature and RH (∼22 °C and 48%). Heat treatment should inactivate antiviral enzymes, proteins, and bacteria.^23^ By investigating virus inactivation in bulk, we could isolate the effects of solution composition from other factors such as evaporation.

**Figure 3:**
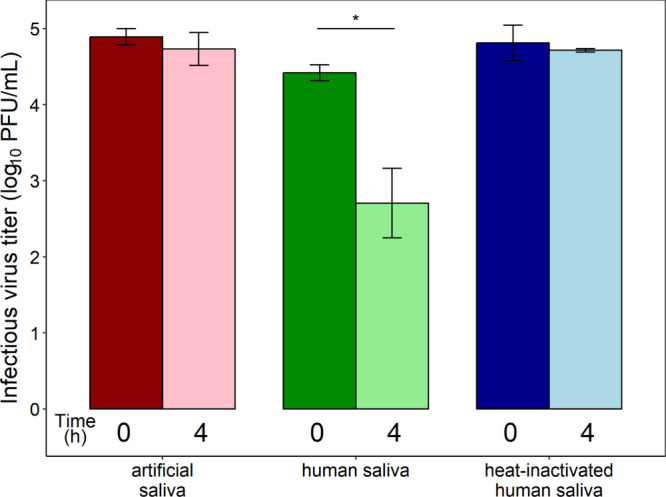
Infectious virus titer (log_10_ PFU/mL) of H1N1pdm09 IAV in bulk media (500 μL) at 0 and 4 h. Each bar is the average ± standard deviation of three replicates.

We observed significant loss of IAV infectivity in bulk human saliva over 4 h; however, we observed no significant loss of infectivity in either artificial or heat-inactivated saliva (Figure 3). This result suggests that biological components present in human saliva contribute to inactivation of IAV. Interestingly, our results contrast with those of Rockey et al.,^10^ who did not observe substantial decay of H1N1pdm09 or H3N2 (another IAV strain) in the same type of saliva over a period of 2 h. This difference may reflect the delayed onset of antiviral activity.^54^ Kong et al.^51^ recently observed ∼1.5log_10_ loss of titer over 8 h in 2 μL droplets of heat-inactivated human saliva supplemented with 0.1 mg/mL lysozyme.

This experiment suggests that while studying virus inactivation in surrogate fluids is valuable for deciphering mechanisms of inactivation, results may overestimate virus stability compared to that in physiological fluids and may neglect the impact of (bio)­chemical factors on decay. In real-world transmission scenarios, antiviral components of respiratory fluids appear to accelerate virus inactivation, potentially reducing the role of other factors. Future studies should investigate the impact of biological components on virus inactivation in droplets and should include evaporation and other factors that may affect the process.

## Limitations and Future Studies

One limitation of this study is our use of sessile, 1 μL droplets on polypropylene surfaces to investigate virus inactivation. This scenario, while relevant to fomite transmission, cannot replicate the physical and chemical processes occurring within smaller, suspended aerosol droplets. Sessile, 1 μL droplets have a smaller surface-area-to-volume ratio and evaporate more slowly compared to respiratory droplets that may be as much as 10^10^ times smaller in volume. Furthermore, reaction rates, including potential pH changes, may differ between large and small droplets since the time scale of diffusive processes scales with the droplet radius squared.^55^ Micron-scale droplets evaporate within seconds, potentially limiting the extent of pH change before the droplets equilibrate with the gas phase and the pH stabilizes. While we measured a slight shift in pH, the extent of this shift and its translation to other respiratory fluids (i.e., airway surface liquid or nasal mucus) in the presence (or absence) of trace gases, remain unclear. Additionally, our methods were not able to detect rapid changes in virus stability and pH that have been reported in other studies.^8,14^

To our knowledge, the measurement of pH in highly complex, partially crystallized droplets has not been attempted previously, and it presented unique challenges and limitations. As a highly nonideal solution, saliva exhibits strong ion–ion interactions. Under such conditions, appropriate estimates of ion (e.g., H^+^) activity coefficients are challenging and may hinder accurate pH estimation. Further, the dissociation of 4-MBA on the pH nanoprobes may be influenced by low water activity, potentially resulting in misinterpretation of the collected spectra. Despite uncertainty around the absolute pH values, the relative differences between the pHs at high RH and the same atmospheric conditions are more certain. Further research should aim to optimize nanoprobe-based sensing, particularly for supersaturated and partially crystallized aerosol particles and smaller droplets.

Due to experimental time constraints, no data are available for the wet phase of the droplets at 30% RH. Decay kinetics differ between the wet and dry phases of the droplets.^1,10^ Previous studies have shown a linear increase in decay over time during the wet phase,^27^ including for IAV suspended in the same saliva as used in this study.^10^ We hypothesize that a similar relationship existed in our study, but future studies should focus on decay kinetics over shorter time intervals to capture differences between the wet and dry phases.

Our infectivity results are limited to human saliva, which is only one of several types of respiratory fluid that may serve as vehicles for transmission. Saliva varies in chemical and biological composition from person to person (Tables S3, S4, and S8) and from other respiratory fluids (e.g., airway surface liquid, nasal mucus). Thus, we recommend caution in translating results between respiratory fluids.^10^ Another limitation of our study is the ambiguity of “ambient air.” Undetected trace gases can potentially influence virus inactivation. However, our experiments were conducted within a biosafety cabinet that filters incoming air, greatly reducing the concentrations of “sticky” gases with high dry deposition velocities, such as nitric acid and ammonia. Multiple interacting factors appear to drive virus decay, and it is probable that unknown factors and their interactions still need to be identified before we can fully comprehend the mechanisms of virus inactivation in aerosol particles and droplets.

## Supplementary Material



## Notes

Additional methodological details, environmental conditions in the chamber during experiments (Figure S1, S2), composition of artificial saliva (Table S1), Raman spectra of polystyrene and quartz (Figure S3), droplet evaporation curves on polystyrene and quartz (Figure S4), environmental conditions during pH measurements (Table S2), pH calibration curves (Figures S5), pH in droplets using dry calibration curve (Figure S6), bulk and droplet pH measurements as a function of MEM:saliva ratio (Figure S7), inorganic ion concentrations in saliva (Table S3), alkalinity titration data (Table S4), droplet drying times (Table S5), environmental conditions during experiments with Phi6 (Figure S8), decay of Phi6 (Figure S9), MINEQL+ modeling results (Table S6, S7, and S9), alkalinity of saliva and media (Table S8), virus decay in the presence of supplemental bicarbonate (Figure S10), and buffer capacity (Figure S11) (PDF)
