# Supplementary material for: Influence of Carbon Dioxide and pH on Influenza Virus in Sessile Saliva Droplets
Source: Environ Sci Technol. 2026 Feb 26;60(9):7274–82. doi: 10.1021/acs.est.5c12672 (PMC12980841; doi:10.1021/acs.est.5c12672)
Supplement: Supplementary file 1 [file es5c12672_si_001.pdf]

Supplemental Information

Influence of Carbon Dioxide and pH on Influenza Virus in Sessile Saliva Droplets

Alexandra K. Longest[1], Sonali Srivastava[1], Frank A. Mazzola[1], Rania E. Smeltz[1], Jeffrey L. Parks[1], Liviana K. Klein[1], Nisha K. Duggal[2], Peter J. Vikesland[1,*], and Linsey C. Marr[1,*]

[1]Department of Civil and Environmental Engineering, Virginia Tech, Blacksburg, Virginia, USA

[2]Department of Biomedical Sciences and Pathology, Virginia-Maryland College of Veterinary Medicine, Virginia Tech, Blacksburg, Virginia, USA

[*]Corresponding authors: Peter J. Vikesland (vikesland@vt.edu) and Linsey C. Marr (lmarr@vt.edu)

This file includes:
21 pages
Supporting methods
Figures S1 to S11
Tables S1 to S9
Supporting references

Supplementary Methods

Biohazard information.

Experiments using influenza A virus H1N1pdm09 were carried out under biosafety level 2 (BSL2) conditions, with investigators wearing appropriate personal protective equipment. The PPE included lab coat and gloves. All work was in compliance with the Virginia Tech Institutional Biosafety Committee's approved protocols.

IAV stock propagation.

IAV virus stock was grown as described in Pan et al.[1] Briefly, virus propagation was conducted by growing 1:50,000 CP1 virus stocks on confluent Madin-Darby canine kidney (MDCK) cells, kindly provided by Dr. Seema Lakdawala, in MEM containing 1% antibiotic-antimycotic (Thermo Fisher, Cat. No. 15240062), 1% L-glutamine, and 1 µg/mL TPCK trypsin (Thermo Fisher, Cat. No. 20233) at 37°C. Virus was harvested after significant cytopathic effect was observed. Cellular material was removed through centrifugation at 200×g for 10 min. Virus stocks were stored in aliquots at -80°C until use.

Influenza A virus (IAV) plaque assays.

IAV samples were quantified by plaque assay as described in Lowen et al.[2] Briefly, 10-fold serial dilutions of the collected samples were prepared. Two-hundred microliter volumes of the serial dilutions in phosphate buffered saline (PBS; Fisher Scientific, Cat. No. 14190144) were deposited over a layer of MDCK cells in a 6-well plate. Plates were incubated for 1 h at 37°C and 5% carbon dioxide (CO_2). The virus suspension was aspirated off, and an agar layer was deposited over the cells. The agar layer consisted of 0.64% agar (Fisher Scientific, Cat. No. LP0028) and 2× MEM infection medium. The 2× MEM medium was composed of 10× MEM (Thermo Fisher, Cat. No. 11430030), double-distilled water, L-glutamine, 7.5% sodium bicarbonate (Thermo Fisher, Cat. No. 25080094), HEPES buffer (Thermo Fisher, Cat. No. 15630080), penicillin-streptomycin (Pen/Strep) (Gibco, Cat. No. 15140122), and 35% BSA (Fisher Scientific, Cat. No. SH3057402). Plates were incubated at 37°C and 5% CO_2 for 48 h and then stained with crystal violet. The number of plaques was counted, and the infectious virus titer in the samples was calculated accordingly.

The change in infectious titer over time was expressed as infectious virus decay ($\log_{10}$ PFU/mL) and was calculated according to **eq. 1**:

$$infectious\ virus\ decay\ (\log_{10} PFU/mL) = \log\frac{N(0)}{N(t)} \qquad \text{(eq. 1)}$$

where $N(0)$ is the infectious titer at time zero and $N(t)$ is the infectious titer at time t. Calculating virus decay by comparing titers at two different time points should account for potential losses due to resuspension.

Phi6 stock and quantification.

Phi6 was propagated in Luria-Bertani medium from stock suspensions according to established methods.[3] Phi6 was ultracentrifuged and resuspended in Dulbecco's modified Eagle medium (DMEM; Thermo Scientific, Cat. No. 11995073) using an Optima XPN-100 centrifuge as previously described.[4] Stocks were diluted to 10^6 PFU/mL for use. Virus titers were quantified by plaque assay.[5] A previous study has demonstrated no difference in decay between virus diluted in DMEM vs. ultracentrifuged and resuspended in DMEM.[6]

Briefly, 10-fold serial dilutions of the collected samples were prepared. Fifty microliters of the serial virus dilutions, 200 µL of liquid culture of *Pseudomonas syringae*, and 4.5 mL of LB soft agar were mixed and poured over plates. Plates were incubated at 25°C for 24 h. After incubation, the number of plaques on the plates was counted, and the infectious titer in the samples was calculated accordingly. Infectious virus decay ($\log_{10}$ PFU/mL) was calculated using **eq. 1**.

IAV environmental data.

Sensors (HOBO UX100-011 and Aranet4 HOME) inside the atmospheric chamber recorded relative humidity (RH), temperature, and CO_2 concentrations for the ambient air (0.04% CO_2) and low CO_2 atmospheres. For the high CO_2 atmosphere, a sensor with a larger range (SAN-10 Personal CO_2 Safety Monitor) was used. The temperature ranged between 21 and 24°C in all experiments, and RH was within ±4% of the targeted value. For low CO_2, the levels in the chamber never exceeded 0.005% (50 ppmv). For high CO_2, the levels ranged between 4.3-5% (43,000 to 50,000 ppmv); they decreased over time perhaps due to small leaks in the chamber. **Figure S1** shows time series of relative humidity (RH), temperature, and CO_2 during the IAV viability experiments. A battery-powered fan inside the chamber enhanced air mixing and accelerated establishment of equilibrium conditions.

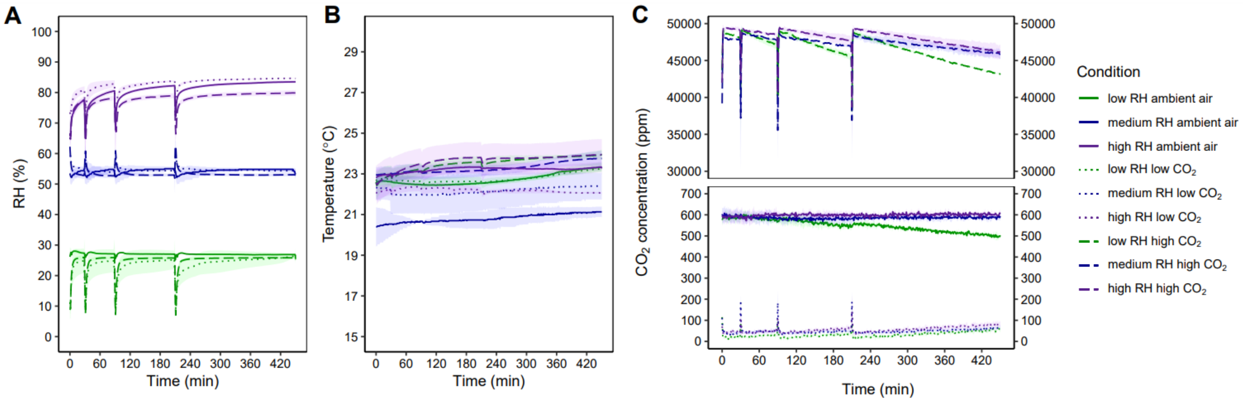

Figure S1. Environmental conditions surrounding H1N1pdm09 droplets were within 4% of targeted RH and maintained temperatures between 20 and 24°C under ambient air (0.04% CO_2), low CO_2 (<0.005% CO_2 and >99.995% N_2), and high CO_2 (4.3-5% CO_2 and >95% N_2). (A) RH, (B) temperature, and (C) CO_2 concentration within the desiccator were recorded every minute during the stability experiments at low (30%), medium (55%), and high (80%) RH. The lines represent the average of three independent replicates, and the shaded areas represent the standard deviations. The legend shows the corresponding color and line type for each RH and atmospheric

condition. The solid lines represent ambient air, the dashed lines represent low CO_2, and the dot-dash lines represent high CO_2. The colors correspond to the targeted RH levels of low (green), medium (blue), and high (purple) RH. Sharp changes in RH and CO_2 reflect opening and closing of the chamber for sample access.

As described in the main paper, an additional viability experiment was conducted by adding sodium bicarbonate ($NaHCO_3$) into human saliva. Following $NaHCO_3$ addition, the solution was vortexed at medium intensity for 5 s to ensure mixing. IAV was diluted into the solution, vortexed again, and then droplets were deposited and resuspended as described previously. These experiments were conducted at medium RH (55%) in low (<0.005% CO_2 and >99.995% N_2) and high CO_2 (4.3-5% CO_2 and >95% N_2). For each experiment, one set of droplets consisted of saliva plus $NaHCO_3$, and one set was a control without additional $NaHCO_3$. Experiments at each atmospheric condition were conducted in independent triplicates.

Figure S2 shows time series of RH, temperature, and CO_2 during the virus inactivation kinetic experiments with and without addition of $NaHCO_3$.

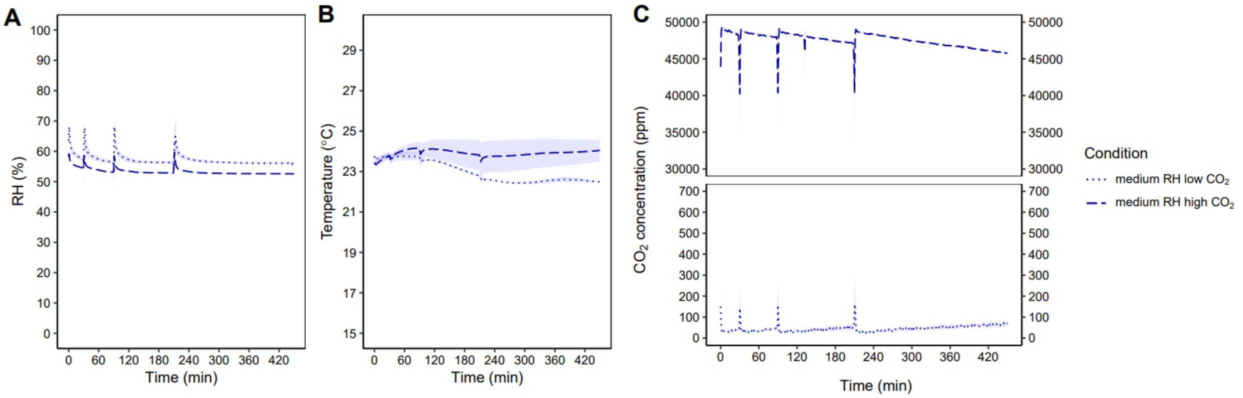

Figure S2. Environmental conditions surrounding H1N1pdm09 droplets with and without sodium bicarbonate were within 5% of targeted RH and maintained temperatures between 22 and 25°C under low CO_2 (<0.005% CO_2 and >99.995% N_2) and high CO_2 (4.3-5% CO_2 and >95% N_2). (A) RH, (B) temperature, and (C) CO_2 concentration within the desiccator was recorded every minute during the stability experiments at medium (55%) RH. The lines represent the average of three independent replicates, and the shaded area represents the standard deviation. The legend shows the corresponding color and line type for each corresponding RH and atmospheric condition. A dashed line represents low CO_2, and the dot dash line represents high CO_2. The color corresponds to the targeted RH levels of medium RH (blue). Sharp changes in RH and CO_2 reflect opening and closing of the chamber for sample access.

Respiratory and surrogate fluids.

Human saliva was purchased from Innovative Research. Saliva collection and preparation methods have been previously described.[7] According to the supplier, individual saliva samples were collected and stored at -80°C. These samples were then pooled by thawing and combining them. The pooled solution was passed through cheese cloth to filter out large particles. Final aliquots were stored at -80°C until purchase. The number of saliva donors and information about them were not disclosed.

Artificial saliva was made using a previously described recipe[8] to replicate that used in other studies on viral stability in levitated droplets.[9] The composition of artificial saliva is provided in Table S1.

Table S1. Artificial saliva composition in 979 mL of DI water

Chemical species	Amount
$MgCl_2 \cdot 7H_2O$	0.04 g
$CaCl_2 \cdot H_2O$	0.13 g
$NaHCO_3$	0.42 g
0.2 M KH_2PO_4	7.70 mL
0.2 M K_2HPO_4	12.30 mL
NH_4Cl	0.11 g
KSCN	0.19 g
$(NH_2)_2CO$	0.12 g
NaCl	0.88 g
KCl	1.04 g
mucin	3.00 g
DMEM	1.00 mL

Measurement of pH in droplets using Au-nanoparticles and Raman spectroscopy.

Materials.

4-mercaptobenzoic acid (4-MBA), sodium citrate dihydrate ($Na_3Citrate \cdot 2H_2O$), and tetrachloroauric (III) acid ($HAuCl_4 \cdot 3H_2O$) were purchased from SigmaAldrich. Thiolated poly(ethylene) glycol (HS-PEG, 5kDa) was purchased from Nanocs.

pH probe synthesis.

The SERS pH nanoprobes were composed of gold nanoparticles (AuNPs) functionalized with 4-MBA and stabilized with HS-PEG. 4-MBA functionalized nanoprobes are widely used for pH monitoring due to the pH sensitivity of the 4-MBA carboxylate group.[10] Protonation or deprotonation of this group leads to changes in the intensity of the SERS peaks, thus enabling detection of pH.

AuNPs were synthesized using a seed-mediated method, resulting in an average size of 34.5 ± 1.2 nm.[11] SERS pH nanoprobe production was adapted from the protocol previously described by Wei et al.[12] In summary, 990 μL of AuNPs was mixed with 10 μL of 1 mM 4-MBA and vortexed for

10-15 s. The mixture was allowed to sit for 5 min before adding 50 μL of HS-PEG (50 μM). The mixture was vortexed for 10-15 s, allowed to sit for an additional 5 min, and then centrifuged at 7500 rpm for 3 min. The resulting pellet was washed with deionized water to remove unbound reagents. This washing process was repeated twice before the nanoparticles were resuspended and concentrated in MEM solution (75× concentration).

The SERS nanoprobe suspension was combined with purchased human saliva in a 1:5 volume ratio. In the virus viability experiments, a 1:10 dilution of virus in MEM to saliva was used. However, this dilution resulted in Raman signals that were too weak to detect.

Environmental conditions.

Quartz coverslips were placed on top of a layer of aluminum foil (smoothed using a Kimwipe) in a polystyrene petri dish (Corning, Cat. No. 430588). The polystyrene petri dishes were used to transfer samples in and out of the Atmosbag chamber without disturbing the droplets. As noted in the main paper, polystyrene could not be used for the droplet pH measurements since the polymer background signal interfered with the 4-MBA signal (**Figure S3**).

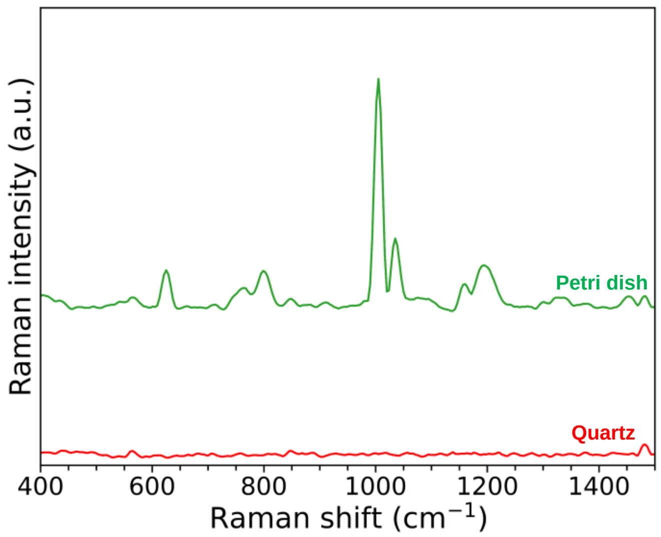

Figure S3. Raman spectra of polystyrene petri dish and quartz. Polystyrene is challenging to use in Raman spectroscopy experiments because it elicits background signals that interfere with the 4-MBA nanoprobe signal. Quartz has minimal background signal.

Droplet evaporation kinetics.

Figure S4 shows the evaporation kinetics of saliva droplets on quartz compared to polystyrene. The droplets evaporate slightly faster on quartz, likely due to its greater hydrophobicity that promotes spreading of the droplets on the surface (i.e., lower contact angle, flatter droplets, and greater surface area for evaporation). This results in a shorter "drying time" and an earlier

stabilization of droplet pH. However, we do not believe this significantly affected results, as the observed changes in pH were relatively minimal.

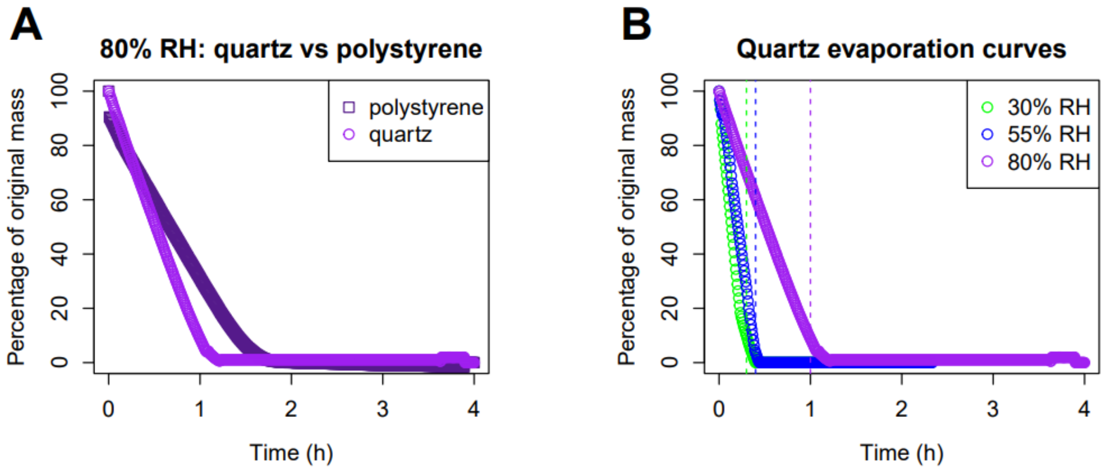

Figure S4. Evaporation curves for ten 1 µL (initial volume), sessile saliva droplets. (A) Saliva droplets deposited on quartz (dark purple) evaporated faster compared to droplets on polystyrene (light purple). (B) Droplet drying time, indicated by the dashed, vertical lines, varied with RH. Technical duplicate measurements of ten 1 µL saliva droplets were performed.

Environmental conditions, including RH, temperature, and CO_2 concentration, were measured in the droplet pH experiments. High CO_2 concentrations were measured using a ToxiRAE Pro CO_2 (Honeywell), as the SAN-10 Personal CO_2 meter that was used in the virus viability experiments was no longer available. RH was maintained using saturated salt solutions as previously described. **Table S2** summarizes the environmental conditions during the droplet pH experiments.

Table S2. Environmental conditions (average ± standard deviation) during droplet experiments to measure pH with SERS

Atmospheric condition	Targeted RH (%)	Measured RH (%)	CO_2 concentration (ppm)	Temperature (°C)
Ambient air	30	28.5 ± 1.0	525 ± 54	22.1 ± 0.2
	55	55.6 ± 0.9	488 ± 19	22.9 ± 0.3
	80	82.1 ± 1.4	596 ± 61	22.3 ± 0.3
Low CO_2 (<0.005% CO_2 and >99.995% N_2)	30	22.2 ± 4.8	14 ± 15	22.3 ± 0.3
	55	58.2 ± 2.6	4 ± 12	22.4 ± 0.3
	80	83.9 ± 4.8	28 ± 27	22.6 ± 0.3
High CO_2 (4.3-5% CO_2 and >95% N_2)	30	26.0 ± 1.4	44050 ± 2058	23.1 ± 0.4
	55	57.3 ± 1.6	44750 ± 475.2	22.5 ± 0.2
	80	78.5 ± 4.1	44500 ± 3874	22.6 ± 0.3

To achieve the desired atmospheric conditions, gas flowed into the Atmosbag at 15 psi until it was full, and the gas then was expelled and the bag refilled. This process was repeated until the experimental conditions were met (i.e., <0.005% for low CO_2 and 4.3-5% CO_2 for high CO_2). For medium and high RH, the gas was humidified before entering the chamber. One-microliter droplets were deposited onto a quartz cover slip and left exposed in an open petri dish within the Atmosbag until the designated time had elapsed. Afterwards, the petri dish was covered and quickly removed from the Atmosbag for pH measurements. For the 0.5 and 1 h time points, droplets were deposited in 3-min intervals to account for the 3 min required to scan a single droplet. For the 2 and 4 h time points, all three droplets were deposited sequentially. Previous experiments demonstrated that the droplets were dry by the elapsed time (2 and 4 h), and the pH remained stable during the scanning period. For the measurements at 0 h, droplets were deposited outside the bags and scanned immediately to replicate the conditions of the virus viability experiments.

Instrumentation and data analysis.

Each 1 µL droplet was analyzed using a confocal Raman microscope (Alpha500R, WITec, Germany) equipped with a 10× objective lens and a 785 nm diode laser. Raman scanning was performed using a motorized stage with a lateral (X-Y) travel range of 150 nm ×100 nm and a depth (Z) travel range of 30 mm, with a minimum step size of 10 nm. Measurements were taken at two locations within each droplet: the center and the edge. Each scan covered a 30×30 μm^2 area using a 10×10 grid of 100 data points. The average ± standard deviation of all data points is included in **Figure 2**. The laser was operated at 20 mW with an acquisition time of 0.1 s per point. Instrument calibration was verified prior to measurement using the silicon peak at 520 cm^{-1}. Spectral processing, including cosmic ray removal, Savitzky-Golay smoothing, and baseline subtraction, was carried out using WITec Project Five software (v.5.2).

To measure pH using the SERS probe, a calibration curve was developed based on ratiometric analysis of the intensities at 1425, 1076, and 1710 cm^{-1} (I_{1425}, I_{1076}, *and* I_{1710}).[10,12] The peak ratio (I_{1425}/I_{1076}) for each droplet was plotted against the bulk pH, as measured by a pH meter (Thermo

Scientific, Orion Versa Star Pro pH meter, relative accuracy ±0.002), assuming that the pH of the bulk and the droplets is the same. To test whether increased salt concentration in the droplets would alter the pH measurements, we measured the pH of bulk MEM and saliva (volume ratio of 1:5) and NaCl saturated MEM and saliva solutions (water activity = 0.75, corresponding to deliquesced droplets in equilibrium with 75% RH or a salt concentration (6.1 mol NaCl/kg H_2O) of the remaining organic rich solution after efflorescence in equilibrium with the NaCl crystal[13]). We observed no difference in pH; therefore, we assumed that a difference between pH in bulk and in 1 µL droplets, which are not supersaturated, in our calibration curves would not occur due to increased salt concentration.

The bulk pH was adjusted with HCl (1 M) for values below the initial pH (~8.5) and with NaOH (1 M) for values above. Calibration curves for the pH nanoprobes in MEM and saliva (volume ratio of 1:5) were obtained using a Boltzmann equation fit. Two calibration curves were generated for both wet and dry, effloresced droplets at ambient room temperature (~22°C) and RH (48%) as shown in **Figure S5**. The distinction between wet and dry droplets was based on their RH-dependent drying times and the observation that the droplets evaporate more rapidly on quartz (**Figure S4A**). For wet conditions, droplets were deposited on the quartz at room RH and temperature and immediately scanned (<3 min) to allow for as little as possible evaporation during this time period. For the 0 h time point for all RH conditions, the pH of the droplets was estimated using the wet droplet calibration curve. At 0.5 h for high RH, the droplets were not fully dry, but some evaporation had occurred. Thus, points were plotted using both the wet and dry calibration curves to show the range of potential pH values. For subsequent time points, the dry droplet calibration curve was applied. As shown in **Figure S4A**, the droplets reached quasi-equilibrium by 1 h at high RH; thus, the dry droplet calibration curve was utilized for the 1, 2, and 4 h time points at high RH. Droplets were dry by 0.5 h at low and medium RHs (**Figure S4B**) and thus the dry droplet calibration curve was used for those conditions.

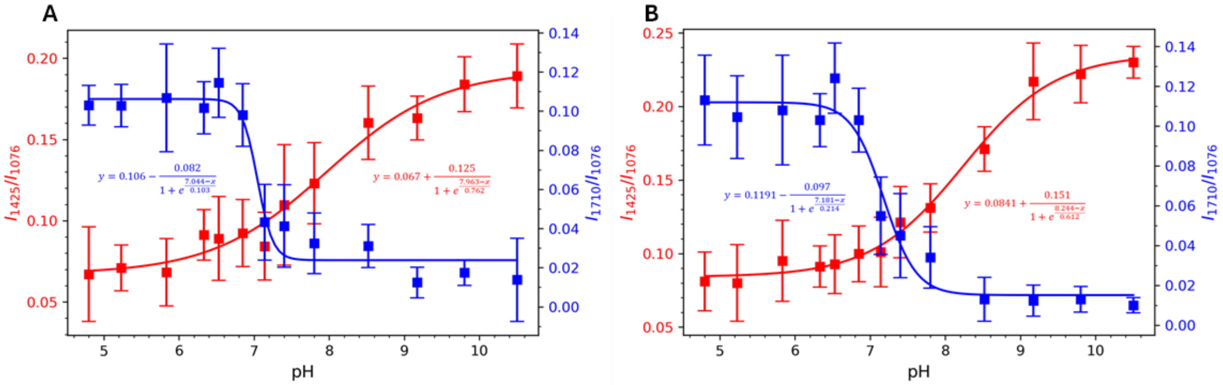

Figure S5. pH Calibration curves constructed by curve fitting ratios of I_{1425}/I_{1076} and I_{1710}/I_{1076} vs. the solution pH and their fit using the Boltzmann equations for (A) wet, and (B) dry droplets of pH probe in a nanoparticle suspension consisting of MEM and saliva (volume ratio of 1:5). These were conducted at room temperature (~22°C) and RH (48%).

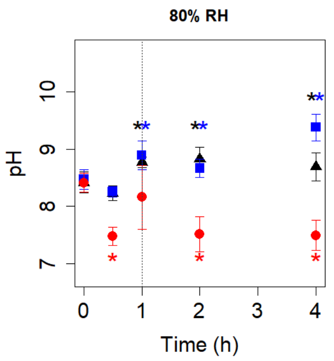

Figure S6. pH of 1 µL (initial volume), sessile, evaporating saliva droplets in ambient air (~0.04% CO_2), low CO_2 (<0.005% CO_2 and >99.995% N_2), and high CO_2 (4.3-5% CO_2 and >95% N_2) at 80% RH after 0, 0.5, 1, 2, and 4 h using dry calibration curve for 0.5 h. Each point represents the average ± standard deviation of two independent replicates with three independent technical replicates. Asterisks indicate significant differences between the pH at 0 h and a subsequent time point. Dotted, grey, vertical lines represent droplet drying times on quartz (Figure S4B).

Effect of saliva concentration on bulk and droplet pH using SERS.

We measured both the bulk pH and the droplet pH at varying MEM:saliva ratios to evaluate the impact of saliva concentration on pH nanoprobe response. The volumetric ratios used were 1:5, 2:5, 1:1, and 2:1, corresponding to saliva concentrations of 83.3%, 71.4%, 50.0%, and 33.3%, respectively. Bulk pH was determined using a pH probe, while droplet pH was measured using nanoprobes and our SERS calibration curves. As the saliva concentration changed by 60%, the measured change in pH was less than 5% and not statistically significant (**Figure S7**). Changing the percentage of saliva alters the concentration of ions and background proteins and such changes could have adversely affected the pH measurements; however, we did not observe such an effect.

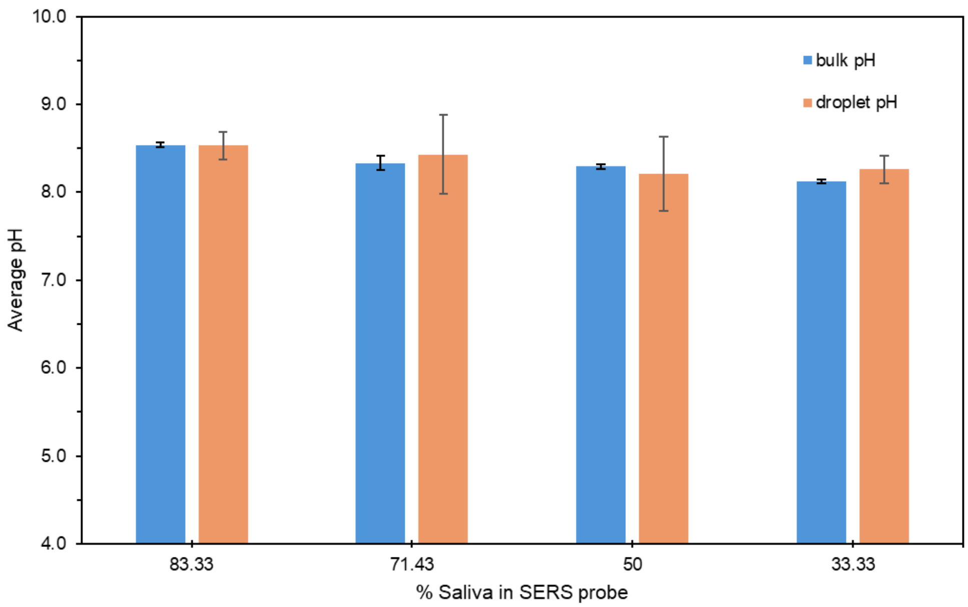

Figure S7. Average bulk and droplet pH measurements of the SERS nanoprobe at varying saliva concentrations: 83.33% (used in this study), 71.43%, 50%, and 33.33%. Bars represent the average ± standard deviation.

Inorganic ion analysis.

Inorganic cation and anion concentrations, measured by inductively coupled plasma mass spectrometry (ICP-MS), for the purchased human saliva and that pooled from three individuals are shown in **Table S3**.

Table S3. Inorganic ion concentrations in human saliva

Element[1]	Symbol	Concentration (mg/L)	
		**Purchased saliva**	**Saliva from three individuals, pooled**
Potassium	K^{+}	807.17	857.04
Chloride[2]	Cl^{-}	520.00	463.39
Sodium	Na^{+}	213.13	102.13
Phosphorus	P^{3-}	128.36	212.84
Sulfur[2]	S^{2-}	125.00	114.16
Calcium	Ca^{2+}	17.49	39.94
Magnesium	Mg^{2+}	2.44	3.73
Silicon	Si	1.48	2.13
Titanium	Ti^{4+}	0.18	0.30
Zinc	Zn^{2+}	0.05	0.02
Iron	Fe^{2+} or Fe^{3+}	0.02	0.02
Vanadium	V*	0.01	BDL
Strontium	Sr^{2+}	0.01	0.02
Lithium	Li^{+}	0.01	BDL
Copper	Cu^{1+} or Cu^{2+}	BDL	0.01
Cobalt	Co^{3+}	BDL	0.01

[1] Only elements with a concentration greater than 1 ppb are reported

[2] The concentrations of chloride and sulfur are less reliable by ICP-MS

*Vanadium is a transition metal that can have charges ranging from +2 to +5

Alkalinity titration.

The total alkalinity of the saliva samples was determined by titrating bulk saliva samples to a final pH of 4.5 using 0.01894 N sulfuric acid using standard methods.[14] The total alkalinity is calculated using **eq. 2**:

$$Total\ alkalinity\ (N) = \frac{(Volume\ of\ acid\ added) \times (Acid\ normality)}{Sample\ volume} \quad \text{(eq. 2)}$$

Titration results and total alkalinities for purchased saliva and for saliva procured from three individuals are summarized in **Table S4**.

Table S4. Saliva titration summary

Sample	Sample volume (mL)	Initial acid level	Final acid level	Total acid added (mL)	Alkalinity (N)	Alkalinity (mg $CaCO_3$/L)
Subject 1	10	8.84	12.3	3.46	6.55×10^{-3}	328
Subject 2	10	12.3	17.71	5.41	1.02×10^{-2}	512
Subject 3	5	17.74	19.2	1.46	5.53×10^{-3}	277
Purchased saliva	10	19.56	28.02	8.46	1.60×10^{-2}	801

Equilibrium pH calculations.

The equilibrium pH was calculated using MINEQL+ (Version 4.62.3). For this purpose, we selected the system components Cl^-, PO_4^{3-}, SO_4^{3-}, CO_3^{2-}, K^+, Na^+, Ca^{2+}, Mg^{2+}, H^+, and H_2O. These components represent the major inorganic constituents (i.e., with concentrations > 10 µM) measured using ICP-MS (**Table S3**). We assumed that the phosphorous measurements obtained by ICP-MS primarily reflected inorganic phosphate.

Estimation of the alkalinity of DMEM and MEM.

We calculated the total alkalinity of both DMEM and MEM based upon their stated $NaHCO_3$ concentrations. Their pH is near neutral, and the contribution of the ingredient sodium phosphate monobasic to alkalinity is negligible.

For high-glucose DMEM with a $NaHCO_3$ concentration of 3.7 g/L:

$$\frac{3.7\ g\ NaHCO_3}{L} \times \frac{mol}{84.01\ g} = 0.044\ M\ NaHCO_3$$

$$\frac{0.044\ mol\ NaHCO_3}{L} \times \frac{1\ equivalent}{1\ mole} \times \frac{50\ g\ CaCO_3/L}{1\ equivalent/L} \times \frac{1000\ mg}{1\ g} = 2202\frac{mg}{L}\ as\ CaCO_3$$

The same calculation can be made for MEM using its lower $NaHCO_3$ concentration of 2.2 g/L to obtain a final alkalinity of 1310 mg $CaCO_3$/L.

Droplet drying times.

The time to quasi-equilibrium, or "drying time" (t_{dry}) was calculated using data previously published by Rockey et al.[7] They measured the change in droplet mass over time using the same purchased human saliva and droplet sizes as employed herein. Using their data, we plotted drying time against RH and fitted an exponential curve through the data, achieving an R^2 value of 0.998. The estimated drying time as a function of RH was calculated using **eq. 3**:

$$t_{dry} = 0.1489e^{0.0267RH} \quad \text{(eq. 3)}$$

Rockey et al.[7] conducted one set of their experiments at an RH of 80%, the same as used in our experiments, so we used Rockey et al.'s results for drying time at this RH rather than estimating it according to **eq. 3**. **Table S5** shows the estimated drying times for the RHs used in our experiments.

Table S5. Calculated drying times (t_{dry}) for 10 sequentially deposited 1 µL saliva droplets

RH (%)	Drying time (h)
30	0.33
55	0.65
80	1.29

Phi6 stability in varying atmospheric conditions.

The stability of Phi6 suspended in DMEM was measured in a desiccator (Fisher Scientific) used as an atmospheric chamber at low, medium, and high RHs at room temperature in two atmospheric compositions. Saturated salt solutions (potassium acetate, magnesium acetate, and potassium chloride) were used to target 27%, 55%, and 80% RH, respectively.[15] The two atmospheres were ambient air containing 0.04% CO_2 as a baseline and ultra-high purity N_2 (Airgas, Lot No. 72-402796545-1) containing negligible CO_2. The nominal descriptor used for the ultra-purity N_2 is "low CO_2" (<0.005% CO_2 and >99.995%). An Aranet4 HOME sensor placed inside the chamber recorded RH, temperature, and CO_2 concentration. The temperature ranged between 21 and 25°C in all experiments. RH initially dropped rapidly when the gas flow began, due to the low RH of the N_2 gas. However, it returned to the target RH within 10 min and remained stable at the desired level throughout the experiment. A battery-powered fan inside the chamber enhanced air mixing and accelerated establishment of equilibrium. **Figure S8** shows the time series of RH, temperature, and CO_2 during the experiments.

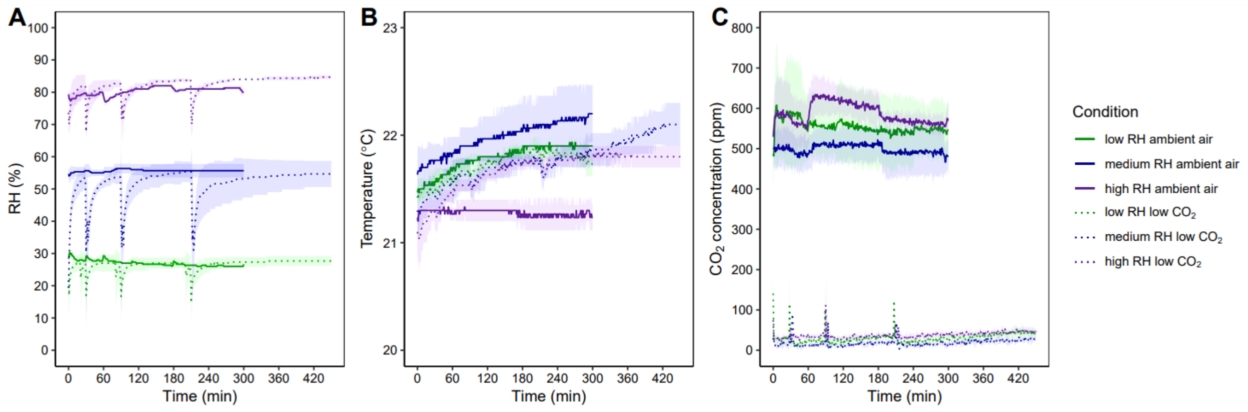

Figure S8. Environmental conditions surrounding Phi6 DMEM droplets under ambient air and low CO_2 (<0.005% CO_2 and >99.995%) conditions. (A) RH, (B) temperature, and (C) CO_2 concentration (C) within the desiccator were recorded every minute during the experiments at low (27%), medium (55%), and high (80%) RH. The lines represent the average of three independent replicates, and the shaded areas represent the standard deviation. The legend shows the corresponding color and line type for each RH and atmospheric condition. A solid line represents ambient air and a dashed line represents low CO_2. The colors correspond to the targeted RH levels of low (green), medium (blue), and high (purple) RH.

For experiments in ambient air, 10 1-µL droplets of Phi6 in DMEM were pipetted onto 6-well polystyrene plates (Corning, Cat. No. 07201588) in technical duplicates. Since the air composition was only minorly affected by opening and closing the chamber, two plates were placed in the chamber simultaneously to increase efficiency. Phi6 is stable in DMEM over extended periods, thus eliminating concerns of bulk virus decay during the experiment. For the low CO_2 experiments, the same methods were employed as described for H1N1pdm09. Droplets were resuspended after each time point in 500 µL of DMEM. A measurement was taken at 0 h to establish the initial titer. Independent triplicates were performed for each atmospheric condition at each RH. Samples were immediately stored at -80°C after collection until quantification by plaque assay.

Figure S9 shows Phi6 virus decay under different atmospheric conditions. Overall, the impact of gas-phase composition on Phi6 decay was RH-dependent. At low RH (**Figure S9A**), we observed similar decay in ambient air and low CO_2. At medium RH (**Figure S9B**), we observed significantly less decay in low CO_2 compared to ambient air at 2 and 4 h. At high RH (**Figure S9C**), Phi6 decayed significantly less in low CO_2 compared to ambient air at 4 h.

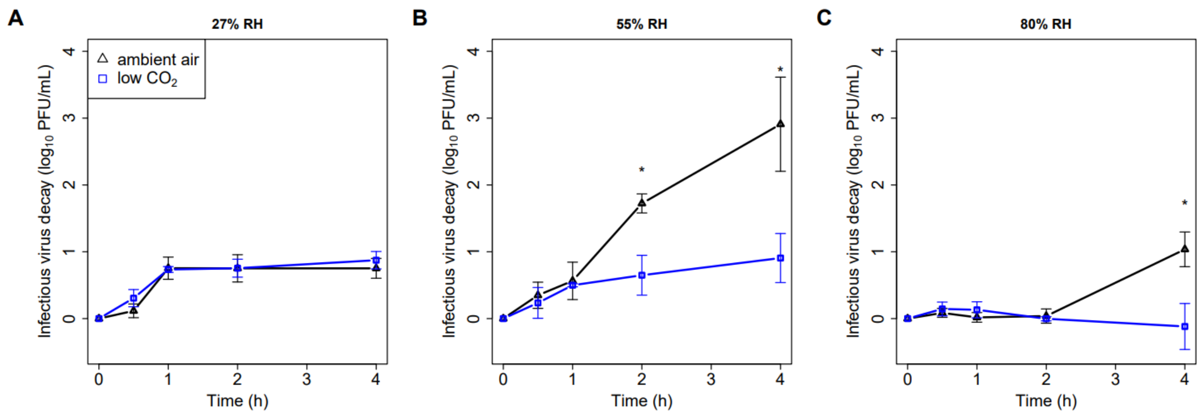

Figure S9. The effect of gas-phase composition on bacteriophage Phi6 decay depends on RH. Titers of Phi6 in ten 1-µL DMEM droplets in ambient air and low CO_2 (<0.005% CO_2 and >99.995%) at (A) 27%, (B) 55%, and (C) 80% RH in terms of $\log_{10}$ decay. Actual RHs were within ± 6% of targeted RHs (**Figure S8**). Each point is the average ± standard deviation of three independent replicates. Asterisks indicate significant differences in atmospheric conditions. Unpaired *t* tests were conducted between samples at each time point to assess significant differences (*$P < 0.05$).

Table S6. MINEQL+ output based upon concentration data in **Table S3**. These calculations assumed an open system, and no restrictions were placed with respect to potential precipitated mineral phases

SYSTEM:	AMBIENT (log CO2 = -3.40)				
MINEQL+ Ver 4.62.3		Page 1			
Data Extracted from : ambient_dropletpH.mdo					
	SINGLE RUN SUMMARY				
This report compiles the output data (concentration, Log C,					
Log K) for all species within a single run.					
~					
MINEQL+	Ver	4.62.3	Page	2	
Data	Extracted	from	:	ambient_dropletpH.mdo	
Run:	1				
	Species		Conc.	Log C	Log K
Type I	COMPONENTS				
	H2O		1.00E+00	0	0
	H(+)		9.77E-10	-9.01	0
	Ca(2+)		3.81E-07	-6.419	0
	Cl(-)		1.47E-02	-1.833	0
	CO3(2-)		8.30E-04	-3.081	0
	K(+)		2.04E-02	-1.69	0
	Mg(2+)		6.96E-05	-4.157	0
	Na(+)		9.09E-03	-2.042	0
	PO4(3-)		1.14E-06	-5.944	0
	SO4(2-)		1.18E-03	-2.928	0
Type II	COMPLEXES				
	OH-	(-1)	1.45E-05	-4.838	-13.85
	CaOH+	(+1)	5.57E-11	-10.254	-12.85
	MgOH+	(+1)	2.03E-07	-6.693	-11.55
	CaHCO3+	(+1)	3.13E-08	-7.505	11
	CaH2PO4+	(+1)	3.16E-11	-10.5	19.88
	CaHPO4	(aq)	4.19E-08	-7.378	13.99
	H2CO3	(aq)	1.36E-05	-4.866	16.24
	HCO3-	(-1)	8.72E-03	-2.059	10.03
	MgHCO3+	(+1)	3.13E-06	-5.504	10.74
	NaHCO3	(aq)	3.17E-05	-4.5	9.63
	KHPO4-	(-1)	7.39E-05	-4.132	12.51
	MgH2PO4+	(+1)	1.24E-08	-7.906	20.22
	MgHPO4	(aq)	1.06E-05	-4.977	14.13
	NaHPO4-	(-1)	5.09E-05	-4.294	12.7
	H2PO4-	(-1)	7.34E-06	-5.134	18.83
	HPO4-2	(-2)	9.44E-04	-3.025	11.93
	H3PO4		7.16E-13	-1.21E+01	20.83
	HSO4-	(-1)	5.69E-11	-10.245	1.69
	CaCO3	(aq)	1.28E-07	-6.894	2.61
	CaPO4-	(-1)	1.61E-07	-6.794	5.57
	CaSO4	(aq)	2.62E-08	-7.581	1.77
	MgCO3	(aq)	1.22E-05	-4.913	2.33
	NaCO3-	(-1)	7.08E-05	-4.15	0.97
	KSO4-	(-1)	8.62E-05	-4.065	0.55
	MgPO4-	(-1)	4.58E-07	-6.339	3.76
	MgSO4 (aq)		3.80E-06	-5.42	1.67
	NaSO4-	(-1)	2.91E-05	-4.537	0.43
Type III	FIXED ENTITIES				
	CO2 (g)				21.1
	H2O				0
Type IV	PRECIPITATED SOLIDS				
	HYDROXYLAPATITE		8.70E-05	0	40.91
Type V	DISSOLVED SOLIDS				
	LIME			-21.246	-3.29E+01
	PORTLANDITE			-11.351	-22.95
	Ca4H(PO4)3:3H2O			-8.706	43.81
	CaHPO4:2H2O			-3.418	17.95
	ARTINITE			-3.719	-10.34
	HYDROMAGNESITE			-8.851	6.24
	PERICLASE			-7.87	-21.73
	BRUCITE			-3.13	-16.99
	Mg(OH)2 (active)			-5.08	-18.94
	MgHPO4:3H2O			-1.977	17.13
	GYPSUM			-5.331	4.02
	NESQUEHONITE			-3.163	4.08
	THERMONATRITE			-8.247	-1.08
	NATRON			-6.299	0.87
	EPSOMITE			-5.553	1.53
	MIRABILITE			-6.343	0.67
	CaHPO4			-3.138	18.23
	ARAGONITE			-1.794	7.71
	CALCITE			-1.614	7.89
	HUNTITE			-3.625	27.59
	DOLOMITE (ordered)			-0.837	15.9
	DOLOMITE (disordered)			-1.387	15.35
	Ca3(PO4)2 (beta)			-4.453	26.69
	ANHYDRITE			-5.581	3.77
	HALITE			-5.626	-1.75
	MAGNESITE			-0.373	6.87
	Mg3(PO4)2			-3.309	21.05
	THENARDITE			-7.779	-0.77
Type VI	SPECIES NOT CONSIDERED				
	pH	(+1)	8.23E-03	-2.084	7
Other Species					
	Activity of H+		8.23E-10	-9.084	0.07

SYSTEM:	low CO2 (log CO2 = -4.52)				
MINEQL+	Ver	4.62.3	Page	1	
Data	Extracted	from	:	lowCO2_dropletpH.mdo	
	SINGLE	RUN	SUMMARY		
This report compiles the output data (concentration, Log C,					
Log K) for all species within a single run.					
~					
MINEQL+ Ver 4.62.3		Page 2			
Data Extracted from : ambient_dropletpH.mdo					
Run: 1					
	Species		Conc.	Log C	Log K
Type I	COMPONENTS				
	H2O		1.00E+00	0	0
	H(+)		1.49E-10	-9.828	0
	Ca(2+)		8.61E-08	-7.065	0
	Cl(-)		1.47E-02	-1.833	0
	CO3(2-)		2.78E-03	-2.556	0
	K(+)		2.04E-02	-1.689	0
	Mg(2+)		5.05E-05	-4.297	0
	Na(+)		8.95E-03	-2.048	0
	PO4(3-)		7.67E-06	-5.115	0
	SO4(2-)		1.18E-03	-2.927	0
Type II	COMPLEXES				
	OH-	(-1)	9.61E-05	-4.017	-13.85
	CaOH+	(+1)	8.21E-11	-10.086	-12.85
	MgOH+	(+1)	9.60E-07	-6.018	-11.55
	CaHCO3+	(+1)	3.49E-09	-8.457	10.99
	CaH2PO4+	(+1)	1.06E-12	-11.975	19.86
	CaHPO4	(aq)	9.22E-09	-8.035	13.97
	H2CO3	(aq)	1.03E-06	-5.986	16.23
	HCO3-	(-1)	4.38E-03	-2.358	10.03
	MgHCO3+	(+1)	1.12E-06	-5.949	10.73
	NaHCO3	(aq)	1.55E-05	-4.808	9.62
	KHPO4-	(-1)	7.30E-05	-4.136	12.5
	MgH2PO4+	(+1)	1.34E-09	-8.874	20.19
	MgHPO4	(aq)	7.46E-06	-5.127	14.11
	NaHPO4-	(-1)	4.95E-05	-4.305	12.69
	H2PO4-	(-1)	1.10E-06	-5.957	18.81
	HPO4-2	(-2)	9.47E-04	-3.023	11.92
	H3PO4		1.63E-14	-13.788	20.81
	HSO4-	(-1)	8.55E-12	-11.068	1.69
	CaCO3	(aq)	9.38E-08	-7.028	2.59
	CaPO4-	(-1)	2.34E-07	-6.631	5.55
	CaSO4	(aq)	5.77E-09	-8.239	1.75
	MgCO3	(aq)	2.88E-05	-4.54	2.31
	NaCO3-	(-1)	2.30E-04	-3.638	0.97
	KSO4-	(-1)	8.52E-05	-4.07	0.55
	MgPO4-	(-1)	2.14E-06	-5.669	3.74
	MgSO4 (aq)		2.69E-06	-5.571	1.65
	NaSO4-	(-1)	2.83E-05	-4.549	0.43
Type III	FIXED ENTITIES				
	CO2 (g)				22.21
	H2O				0
Type IV	PRECIPITATED SOLIDS				
	HYDROXYLAPATITE		8.71E-05	0	40.84
	MAGNESITE		6.31E-06	0	6.85
Type V	DISSOLVED SOLIDS				
	LIME			-20.26	-32.85
	PORTLANDITE			-10.365	-22.96
	Ca4H(PO4)3:3H2O			-9.692	43.74
	CaHPO4:2H2O			-4.075	17.93
	ARTINITE			-1.853	-10.36
	HYDROMAGNESITE			-5.867	6.19
	PERICLASE			-6.377	-21.74
	BRUCITE			-1.637	-17
	Mg(OH)2 (active)			-3.587	-18.95
	MgHPO4:3H2O			-2.127	17.11
	GYPSUM			-5.989	4
	NESQUEHONITE			-2.79	4.06
	THERMONATRITE			-7.745	-1.09
	NATRON			-5.797	0.86
	EPSOMITE			-5.704	1.52
	MIRABILITE			-6.365	0.66
	CaHPO4			-3.795	18.21
	ARAGONITE			-1.928	7.69
	CALCITE			-1.748	7.87
	HUNTITE			-2.64	27.54
	DOLOMITE (ordered)			-0.598	15.88
	DOLOMITE (disordered)			-1.148	15.33
	Ca3(PO4)2 (beta)			-4.782	26.64
	ANHYDRITE			-6.239	3.75
	HALITE			-5.636	-1.75
	Mg3(PO4)2			-2.118	21
	THENARDITE			-7.801	-0.78
Type VI	SPECIES NOT CONSIDERED				
	pH	(+1)	1.25E-03	-2.904	7
Other Species					
	Activity of H+		1.25E-10	-9.904	0.08

SYSTEM:	high CO2 (log CO2 = -1.30)				
MINEQL+	Ver	4.62.3	Page	1	
Data	Extracted	from	:	highCO2_dropletpH.mdo	
	SINGLE	RUN	SUMMARY		
This report compiles the output data (concentration, Log C,					
Log K) for all species within a single run.					
~					
MINEQL+ Ver 4.62.3		Page 2			
Data Extracted from : ambient_dropletpH.mdo					
Run: 1					
	Species		Conc.	Log C	Log K
Type I	COMPONENTS				
	H2O		1.00E+00	0	0
	H(+)		9.72E-08	-7.012	0
	Ca(2+)		2.02E-05	-4.694	0
	Cl(-)		1.47E-02	-1.833	0
	CO3(2-)		1.04E-05	-4.982	0
	K(+)		2.05E-02	-1.689	0
	Mg(2+)		8.20E-05	-4.086	0
	Na(+)		9.17E-03	-2.038	0
	PO4(3-)		6.83E-09	-8.165	0
	SO4(2-)		1.18E-03	-2.929	0
Type II	COMPLEXES				
	OH-	(-1)	1.45E-07	-6.838	-13.85
	CaOH+	(+1)	2.98E-11	-10.526	-12.84
	MgOH+	(+1)	2.41E-09	-8.618	-11.54
	CaHCO3+	(+1)	2.10E-06	-5.677	11.01
	CaH2PO4+	(+1)	1.03E-07	-6.989	19.89
	CaHPO4	(aq)	1.37E-06	-5.865	14.01
	H2CO3	(aq)	1.71E-03	-2.766	16.24
	HCO3-	(-1)	1.10E-02	-1.959	10.04
	MgHCO3+	(+1)	4.69E-06	-5.329	10.75
	NaHCO3	(aq)	4.04E-05	-4.394	9.64
	KHPO4-	(-1)	4.51E-05	-4.346	12.52
	MgH2PO4+	(+1)	8.96E-07	-6.048	20.23
	MgHPO4	(aq)	7.65E-06	-5.117	14.15
	NaHPO4-	(-1)	3.13E-05	-4.505	12.71
	H2PO4-	(-1)	4.46E-04	-3.351	18.84
	HPO4-2	(-2)	5.71E-04	-3.243	11.93
	H3PO4		4.35E-09	-8.362	20.84
	HSO4-	(-1)	5.69E-09	-8.245	1.7
	CaCO3	(aq)	8.63E-08	-7.064	2.61
	CaPO4-	(-1)	5.24E-08	-7.281	5.58
	CaSO4	(aq)	1.41E-06	-5.851	1.77
	MgCO3	(aq)	1.84E-07	-6.736	2.33
	NaCO3-	(-1)	9.04E-07	-6.044	0.98
	KSO4-	(-1)	8.68E-05	-4.062	0.56
	MgPO4-	(-1)	3.32E-09	-8.479	3.77
	MgSO4	(aq)	4.55E-06	-5.342	1.67
	NaSO4-	(-1)	2.95E-05	-4.53	0.44
Type III	FIXED ENTITIES				
	CO2 (g)				19.01
	H2O				0
Type IV	PRECIPITATED SOLIDS				
	HYDROXYLAPATITE		8.21E-05	0	40.95
Type V	DISSOLVED SOLIDS				
	LIME			-23.516	-32.85
	PORTLANDITE			-13.621	-22.95
	Ca4H(PO4)3:3H2O			-6.436	43.85
	CaHPO4:2H2O			-1.905	17.97
	ARTINITE			-9.464	-10.33
	HYDROMAGNESITE			-20.065	6.27
	PERICLASE			-11.793	-21.73
	BRUCITE			-7.053	-16.99
	Mg(OH)2 (active)			-9.003	-18.94
	MgHPO4:3H2O			-2.117	17.15
	GYPSUM			-3.601	4.02
	NESQUEHONITE			-4.986	4.08
	THERMONATRITE			-10.135	-1.08
	NATRON			-8.187	0.87
	EPSOMITE			-5.475	1.54
	MIRABILITE			-6.331	0.67
	CaHPO4			-1.625	18.25
	ARAGONITE			-1.964	7.71
	CALCITE			-1.784	7.89
	HUNTITE			-9.263	27.62
	DOLOMITE (ordered)			-2.83	15.92
	DOLOMITE (disordered)			-3.38	15.37
	Ca3(PO4)2 (beta)			-3.696	26.72
	ANHYDRITE			-3.851	3.77
	HALITE			-5.62	-1.75
	MAGNESITE			-2.196	6.87
	Mg3(PO4)2			-7.512	21.08
	THENARDITE			-7.767	-0.76
Type VI	SPECIES NOT CONSIDERED				
	pH	(+1)	8.21E-01	-0.086	7
Other Species					
	Activity of H+		8.21E-08	-7.086	0.07

Table S7. MINEQL+ calculated equilibrium pH values for droplets as a function of CO_2 concentration and the inclusion (or not) of phosphate in the calculation

		Calculated	Calculated
**Condition**	**log CO_2 (atm)**	**Equilibrium pH**	**Equilibrium pH (no P, no S)**
Ambient CO_2	-3.40	9.084	9.216
Low CO_2	-4.52	9.904	10.004
High CO_2	-1.30	7.086	7.251

Table S8. Total alkalinity of saliva and media

**Sample**	**Total alkalinity (N)**
Subject 1 saliva	0.007
Subject 2 saliva	0.010
Subject 3 saliva	0.006
Purchased human saliva	0.016
DMEM[1]	0.044
MEM[2]	0.026

[1]Calculated value based upon the total concentration of bicarbonate (3.7 g/L) in media
[2]Calculated value based upon the total concentration of bicarbonate (2.2 g/L) in media

Table S9. MINEQL+ calculated contributions of carbonate and phosphate to total alkalinity at an equilibrium pH of 9.084

**Alkalinity (eq/L)**				
**pH**	**Proton**	**Carbonate**	**Phosphate**	**Total**
9.084	1.45×10^{-5}	1.04×10^{-2}	9.46×10^{-4}	1.13×10^{-2}
% of Total:	0.13	91.53	8.34	

Virus stability in the presence of added bicarbonate.

Figure S10 shows virus decay over time in saliva with and without added $NaHCO_3$ in low CO_2 and high CO_2 at medium RH. At this RH, virus decayed most rapidly in our experiments (**Figure 1**). In low CO_2, there were no significant differences in virus decay at any time point (**Figure S10A**). In high CO_2, the only significant difference in decay occurred at 0.5 h (**Figure S10B**), and here, the difference was only 0.18-$\log_{10}$.

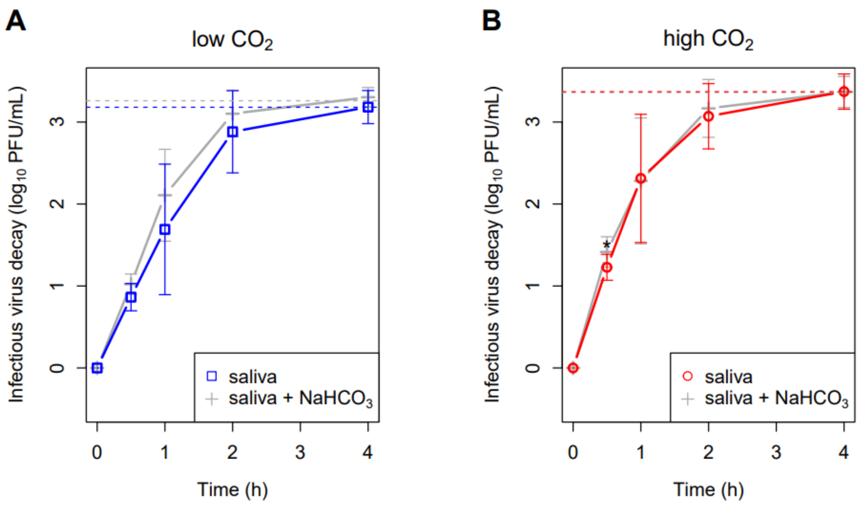

Figure S10. H1N1pdm09 IAV decay in 1 µL (initial volume), sessile, evaporating saliva droplets supplemented with sodium bicarbonate (6.29 µL of 7.5% $NaHCO_3$) in (A) low CO_2 (<0.005% CO_2 and 99.995% N_2) and (B) high CO_2 (4.3-5% CO_2 and >95% N_2) atmospheres at medium RH (55%). Each point is the average ± standard deviation of three independent replicates. Asterisks indicate significant differences between solutions at each time point. Dashed, color-coded lines represent the LOD of each experiment.

A

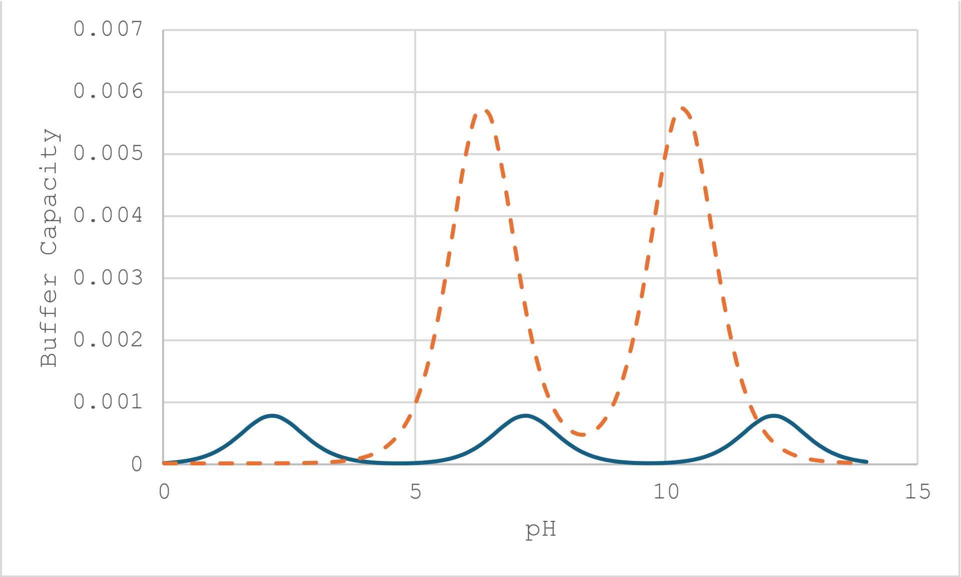

B

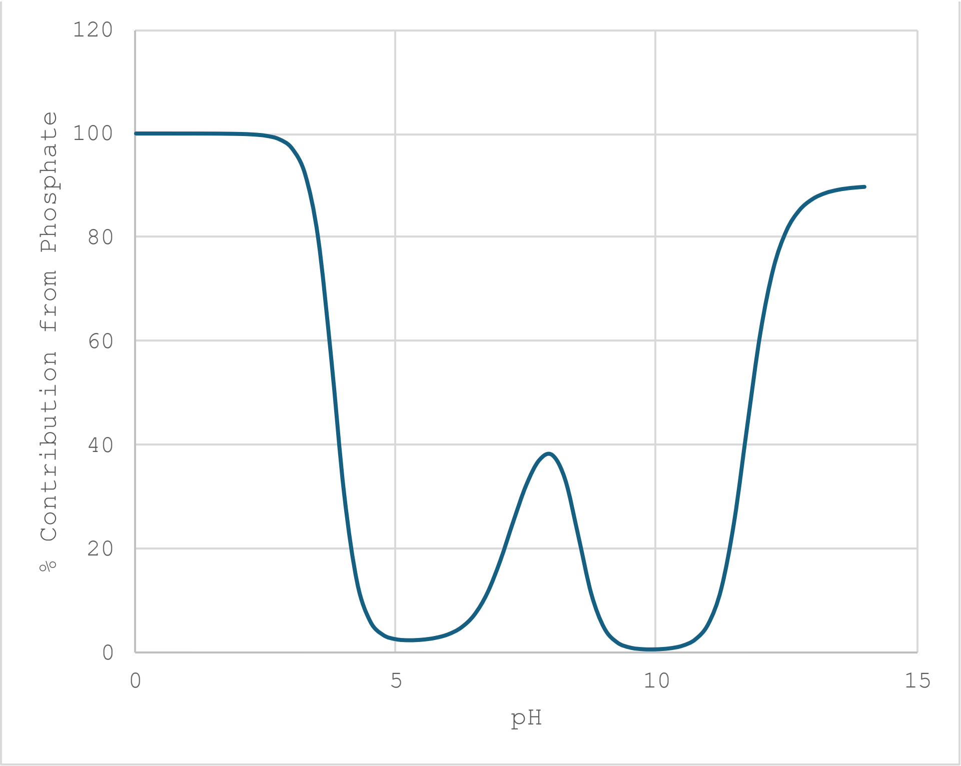

Figure S11. Calculated buffer capacity due to carbonate (0.01 M) and phosphate (0.001 M) species. A) Contributions of carbonate (0.01 M; orange-dashes) and phosphate (0.001 M; blue-solid curves) to buffer capacity. B) Calculated percentage of the overall buffer capacity from phosphate. Buffer capacities were calculated according to Urbansky and Shrock.[16]

Supporting references

1. Pan J, Duggal NK, Lakdawala SS, Rockey NC, Marr LC. Mucin Colocalizes with Influenza Virus and Preserves Infectivity in Deposited Model Respiratory Droplets. Environ Sci Technol. 2025;59(4):2192–200.
2. Lowen AC, Mubareka S, Tumpey TM, García-Sastre A, Palese P. The guinea pig as a transmission model for human influenza viruses. Proc Natl Acad Sci U S A. 2006;103(26):9988–92.
3. Daugelavičius R, Cvirkaitė V, Gaidelytė A, Bakienė E, Gabrėnaitė-Verkhovskaya R, Bamford DH. Penetration of Enveloped Double-Stranded RNA Bacteriophages φ13 and φ6 into Pseudomonas syringae Cells . J Virol. 2005;79(8):5017–26.
4. Kormuth KA, Lin K, Prussin AJ, Vejerano EP, Tiwari AJ, Cox SS, et al. Influenza virus infectivity is retained in aerosols and droplets independent of relative humidity. Journal of Infectious Diseases. 2018;218(5):739–47.
5. Lin K, Marr LC. Aerosolization of Ebola Virus Surrogates in Wastewater Systems. Environ Sci Technol. 2017;51(5):2669–75.
6. French AJ, Longest AK, Pan J, Vikesland PJ, Duggal NK, Lakdawala SS, et al. Environmental Stability of Enveloped Viruses is Impacted by the Initial Volume and Evaporation Kinetics of Droplets. mBio. 2023;14(2):e03452-22.
7. Rockey NC, Sage V Le, Marr LC, Lakdawala SS. Seasonal influenza viruses decay more rapidly at intermediate humidity in droplets containing saliva compared to respiratory mucus. Appl Environ Microbiol. 2024;90(2).
8. Woo MH, Hsu YM, Wu CY, Heimbuch B, Wander J. Method for contamination of filtering facepiece respirators by deposition of MS2 viral aerosols. J Aerosol Sci. 2010;41(10):944–52.
9. Oswin HP, Haddrell AE, Otero-Fernandez M, Mann JFS, Cogan TA, Hilditch TG, et al. The dynamics of SARS-CoV-2 infectivity with changes in aerosol microenvironment. Proc Natl Acad Sci U S A. 2022;119(27):1–11.
10. Wei H, Willner MR, Marr LC, Vikesland PJ. Highly stable SERS pH nanoprobes produced by co-solvent controlled AuNP aggregation. Analyst. 2016;141(17):5159–69.
11. Ji X, Song X, Li J, Bai Y, Yang W, Peng X. Size control of gold nanocrystals in citrate reduction: The third role of citrate. J Am Chem Soc. 2007;129(45):13939–48.
12. Wei H, Vejerano EP, Leng W, Huang Q, Willner MR, Marr LC, et al. Aerosol microdroplets exhibit a stable pH gradient. Proc Natl Acad Sci U S A. 2018;115(28):7272–7.

13. Schaub A, Luo B, David SC, Glas I, Klein LK, Costa L, et al. Salt Supersaturation as an Accelerator of Influenza A Virus Inactivation in 1 µL Droplets. Environ Sci Technol. 2024;58(42):18856–69.

14. APHA. Standard Methods for the Examination of Water and Wastewater. 20th ed. Washington, DC: American Water Works Association and Water Environmental Federation; 1999.

15. Greenspan L. Humidity Fixed Points of Binary Saturated Aqueous Solutions. J Res Natl Bur Stand A Phys Chem. 1977;81A(1):89.

16. Urbansky ET, Schock MR. Understanding, Deriving, and Computing Buffer Capacity. Journal of Chemical Education. 2000;77(12).
